# Tea polyphenol-derived nanomedicine for targeted photothermal thrombolysis and inflammation suppression

**DOI:** 10.1186/s12951-024-02446-z

**Published:** 2024-04-03

**Authors:** Hui Wang, Cui Tang, Yuxia Xiang, Chan Zou, Jianming Hu, Guoping Yang, Wenhu Zhou

**Affiliations:** 1 Center of Clinical Pharmacology, the Third Xiangya Hospital, Central South University, Changsha, Hunan 410013 China; 2 Xiangya School of Pharmaceutical Sciences, Central South University, Changsha, Hunan 410013 China; 3 Academician Workstation, Changsha Medical University, Changsha, 410219 China; 4 NHC Key Laboratory of Prevention and Treatment of Central Asia High Incidence Diseases, Affiliated Hospital, Shihezi University, Shihezi, Xinjiang 832002 China; 5 National-Local Joint Engineering Laboratory of Drug Clinical Evaluation Technology, Changsha, Hunan 410000 China; 6 Hunan Engineering Research Center for Optimization of Drug Formulation and Early Clinical Evaluation, Changsha, Hunan 410013 China; 7 First Department of Pathology, Affiliated Hospital, Shihezi University, Shihezi, Xinjiang Uygur Autonomous Region 832002 China

**Keywords:** Thrombotic diseases, Targeted delivery, Free radical scavenging, Photothermal therapy, Platelet activation, cRGD peptide

## Abstract

Thrombotic diseases impose a significant global health burden, and conventional drug-based thrombolytic therapies are encumbered by the risk of bleeding complications. In this study, we introduce a novel drug-free nanomedicine founded on tea polyphenols nanoparticles (TPNs), which exhibits multifaceted capabilities for localized photothermal thrombolysis. TPNs were synthesized through a one-pot process under mild conditions, deriving from the monomeric epigallocatechin-3-gallate (EGCG). Within this process, indocyanine green (ICG) was effectively encapsulated, exploiting multiple intermolecular interactions between EGCG and ICG. While both TPNs and ICG inherently possessed photothermal potential, their synergy significantly enhanced photothermal conversion and stability. Furthermore, the nanomedicine was functionalized with cRGD for targeted delivery to activated platelets within thrombus sites, eliciting robust thrombolysis upon laser irradiation across diverse thrombus types. Importantly, the nanomedicine’s potent free radical scavenging abilities concurrently mitigated vascular inflammation, thus diminishing the risk of disease recurrence. In summary, this highly biocompatible multifunctional nanomaterial holds promise as a comprehensive approach that combines thrombolysis with anti-inflammatory actions, offering precision in thrombosis treatment.

**Supplementary Information:**

The online version contains supplementary material available at 10.1186/s12951-024-02446-z.

## Introduction

Thrombotic diseases, encompassing conditions like ischemic stroke, myocardial infarction, and venous thromboembolism, pose significant global health threats, resulting in approximately 17.9 million deaths annually [1–3]. These diseases typically arise from factors such as blood vessel damage and the activation of coagulation pathways, ultimately leading to abnormal platelet and fibrinogen aggregation. These processes can result in partial or complete blockage of blood vessels, jeopardizing patients’ lives [4]. Current emergency thrombolytic treatment primarily relies on recombinant tissue plasminogen activator (rt-PA) [4]. However, rt-PA has a short half-life (4–8 min) and can distribute non-specifically throughout the body, potentially causing systemic fibrinolysis and resulting in bleeding complications, thereby limiting its clinical utility [5, 6]. To this end, efforts have been made to devise strategies for targeted drug delivery and controlled release of thrombolytic agents [7, 8]. These approaches, however, often involve intricate preparation procedures and cannot entirely eliminate the risk of bleeding. Therefore, the quest for safer and more effective thrombolytic strategies remains an urgent imperative in clinical thrombosis treatment.

In recent years, there has been a growing interest in the concept of photothermal thrombolysis, a novel approach that harnesses photothermal agents capable of converting near-infrared (NIR) light into thermal energy. This energy is then employed to physically dissolve fibrin clots [9]. Photothermal thrombolysis offers distinct advantages over conventional drug-based methods. It provides site-specific action at the thrombus location in a spatiotemporal manner, thereby reducing the risk of systemic fibrinolysis and associated bleeding complications [10]. Furthermore, the heat generated during photothermal therapy can create microstructural gaps within the thrombus tissue, promoting the enhanced penetration of nanoparticles and augmenting the thrombolysis effect [11]. For instance, a pioneering work demonstrated the capability of gold nanorods irradiated with NIR light to dissolve blood clots, highlighting the potential of this approach [12]. Additionally, Liu et al. explored the application of metal-organic framework-derived carbon nanostructures for dual-modality photothermal/ photodynamic thrombus therapy, achieving an impressive 87.9% recanalization rate in lower limb thrombosis [11]. 

While various materials for photothermal thrombolysis have been reported [11–13], most of these materials are metal-based inorganic compounds and may carry certain biological safety risks. Furthermore, many photothermal thrombolysis approaches primarily target the thrombus itself, overlooking the potential increase in reactive oxygen species (ROS) levels induced by photothermal treatment. Notably, the thrombus microenvironment is characterized by elevated ROS levels due to factors such as hypoxia, vascular endothelial damage, and platelet activation [14]. ROS can exacerbate platelet aggregation, induce inflammatory responses in vascular endothelial cells, worsen vascular conditions, and contribute to restenosis and recurrent thrombosis [15, 16]. Given these considerations, there is a growing interest among clinicians in the clearance of ROS and the inhibition of inflammatory factors in the treatment of thrombotic diseases [17]. However, it is noteworthy that therapeutic strategies addressing these aspects remain underexplored in the current medical literature.

**Scheme 1:**
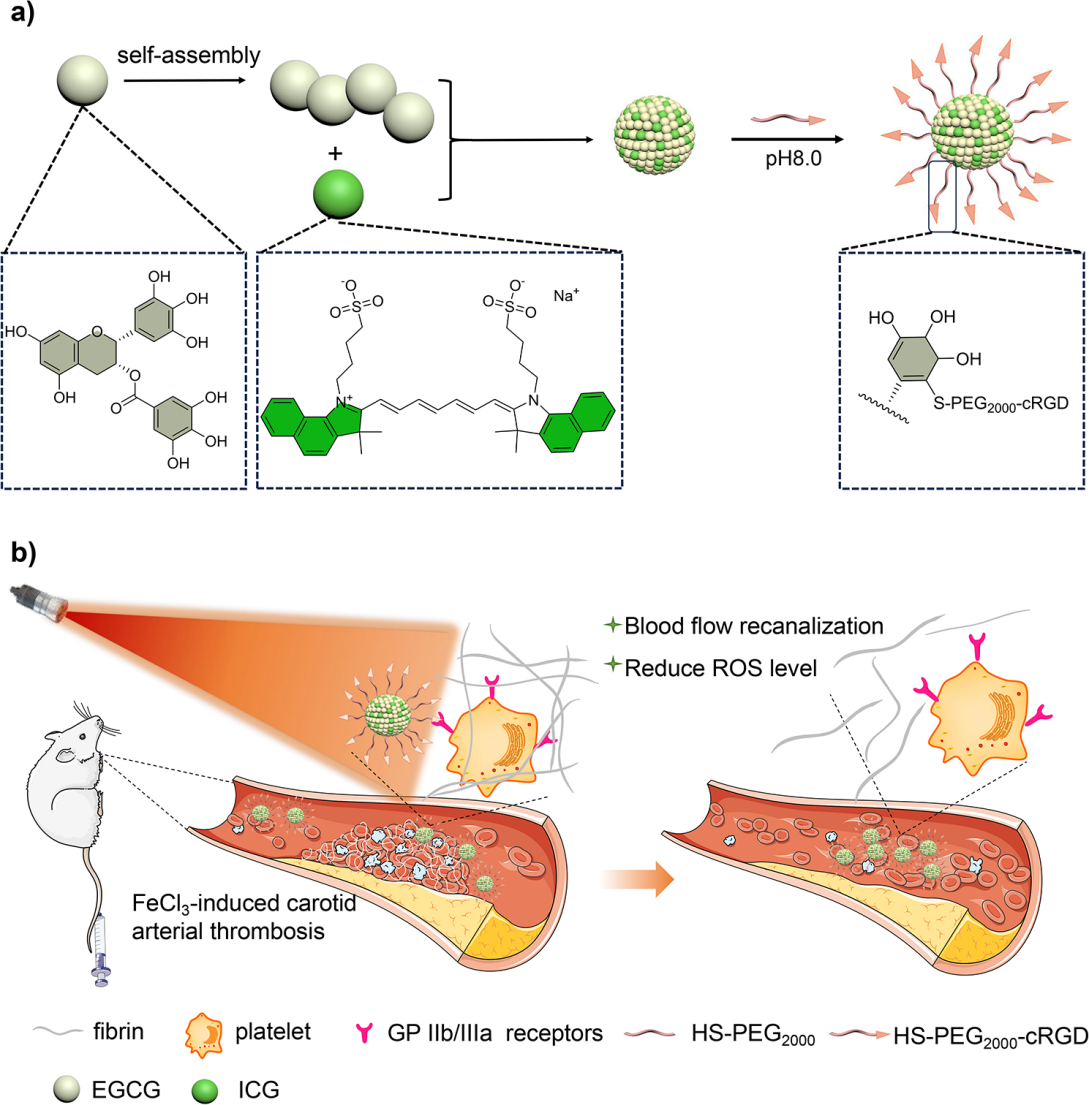
Illustration of (**a)** the preparation of TPNs/ICG-cRGD and (**b)** TPNs/ICG-cRGD mediated specific antithrombotic therapy

In response to these formidable challenges, we introduce a versatile photothermal nanomaterial endowed with free radical scavenging and anti-inflammatory properties, tailored for targeted thrombosis treatment. This nanomaterial originates from Tea polyphenols nanoparticles (TPNs), a subject of our prior research [18], synthesized through the oxidation and self-polymerization of epigallocatechin-3-gallate (EGCG). TPNs have demonstrated remarkable free radical scavenging and anti-inflammatory characteristics, rendering them useful in the treatment of inflammatory disorders [18, 19]. Our study reveals that TPNs inherently possess photothermal capabilities. These capabilities can be significantly augmented by the incorporation of indocyanine green (ICG), an FDA-approved near-infrared photosensitizer, which suffers from several limitations, including poor resistance to photobleaching, short half-life, and lack of specific accumulation in target tissues [20, 21]. EGCG’s unique molecular interactions with ICG facilitate the efficient loading of ICG into TPNs via a straightforward assembly procedure, resulting in TPNs/ICG (Scheme 1) a). This formulation considerably enhances the photostability of ICG, mitigating photobleaching, and engenders a synergistic amplification of the photothermal effect through the cooperation of TPNs and ICG. To enable precise delivery to thrombus sites, the nanoparticle surface is further functionalized with cRGD, a ligand with the capacity to specifically target activated platelets within the thrombus vicinity (scheme 1) b). Upon exposure to laser irradiation, the resultant TPNs/ICG-cRGD exhibit robust thrombolytic potential across various types of thrombi, facilitated by photothermal conversion. Notably, they effectively ameliorate inflammation at the thrombus site without eliciting any untoward toxic effects, thus presenting a novel formulation strategy for photothermal thrombolysis treatment.

## Materials and methods

### Materials, cells and animals

#### Materials

(-)-Epigallocatechin gallate (EGCG, 98%) was acquired from Chengdu Wagott Bio-Tech Co., Ltd. (Chengdu, China), and manganese chloride (MnCl_2_) was obtained from Aladdin (Shanghai, China). HS-PEG-cRGD and HS-mPEG was procured from MeloPEG (Shenzhen, China). ICG and FITC was sourced from Aladdin (Shanghai, China) and J&K Scientific (Beijing, China). 2′,7′-Dichlorodihydrofluorescein diacetate (DCFH-DA) probe was acquired from Thermo fisher scientific. 3-(4,5-dimethyl-2-thiazolyl)-2,5-diphenyl-2 H tetrazolium bromide (MTT) was purchased from Sigma-Aldrich (St. Louis, USA). The Nitric oxide assay kit was purchased from Beyotime (Shanghai China). Inhibition and produce superoxide anion assay kit, hydroxyl free radical assay kit was obtained from Nanjing Jiancheng Bioengineering Institute (China). RPMI 1640 medium, penicillin-streptomycin solution, phosphate buffered saline (PBS), and fetal bovine serum (FBS) were purchased from Gibco Life Technologies, Inc. (Grand Island, NY, USA). Rat, IL-6, IL-8, CRP and TNF-α ELISA kit were purchased from ZCIBIO Technology Co.,Ltd. (Shanghai, China).

#### Cell

Murine macrophage cells (RAW264.7), mouse aortic vascular smooth muscle (MOVAS), human umbilical vein endothelial cells (HUVECs) were obtained from Xiangya Cell Center (Changsha, China). The MOVAS were cultured in DMEM medium, while the RAW264.7 cells and HUVECs were cultured in RPMI 1640 medium, All the culture media were supplemented with 1% penicillin-streptomycin solution and 10% FBS, and the cells were incubated at 37℃ with 5% CO_2_ atmosphere.

#### Animal

Healthy male SD rats (100–130 g) were purchased from Hunan SJA Laboratory Animal Co., Ltd (Changsha, China) and housed in a sterile environment with ad libitum access to food and water. The experimental protocol for animal studies was approved by the Experimental Animal Ethics Committee of Central South University (approval number: CSU-2022-0358) and conducted in accordance with the National Act on the Use of Experimental Animals (People’s Republic of China).

### Preparation of TPNs/ICG-cRGD

ICG (0.129 mM) and EGCG (2.5 mM) were reacted in N-2-hydroxyethylpiperazine-N-ethane-sulphonic acid (HEPES) buffer (10 mM, pH 8.0) for 15 min. Then, 2mM MnCl_2_ and 0.4mM HS-PEG-cRGD were added to the reaction mixture and the reaction was continued for 7 h at 25 ℃. The resulting TPNs/ICG-cRGD were collected by centrifugation at 16,000 rpm for 15 min. The nanoparticles were washed three times with deionized water and stored at 4 ℃ for further use.

### Characterization of TPNs/ICG-cRGD

The morphology of TPNs/ICG-cRGD was observed using transmission electron microscopy (TEM) (Titan G2-F20, FEI, USA). The size distribution and ζ potential were measured in ultra-pure water by using a Malvern zeta Sizer Nano series (Nano ZS, Malvern Instruments, UK). The UV-Vis spectra were detected by UV Spectrophotometer (UV-2450, Shimadzu, Japan). The infrared spectra of the nanoparticles were measured using an infrared spectrometer (Alpha, BRUKE, Germany) to confirm the successful conjugation of HS-mPEG or HS-PEG-cRGD. The grafting rate of HS-mPEG or HS-PEG-cRGD was detected by a sulfhydrylation kit [11]. The entrapment efficiency (EE) and drug loading rate (LR) of ICG were determined by UV Spectrophotometer [22]. The stability of TPNs/ICG-cRGD in various mediums was evaluated by monitoring changes in nanoparticle size.

$$\mathbf{G}\mathbf{R}\left(\mathbf{H}\mathbf{S}-\mathbf{P}\mathbf{E}\mathbf{G}-\mathbf{c}\mathbf{R}\mathbf{G}\mathbf{D}\mathbf{\%}\right)=$$

$$\frac{Total\,sulfhydryl\,content-Sulfhydryl\,content\,in\,the\,supernatant}{Total\,sulfhydryl\,content}$$

$$\mathbf{E}\mathbf{E} \left(\mathbf{I}\mathbf{C}\mathbf{G}\mathbf{\%}\right)=$$

$$\frac{Total\,weight\,of\,added\,ICG-Weight\,of\,ICG\,in\,the\,supernatant}{Total\,weight\,of\,added\,ICG}$$

$$\mathbf{L}\mathbf{R} \left(\mathbf{I}\mathbf{C}\mathbf{G}\mathbf{\%}\right)=$$

$$\frac{Total\,weight\,of\,added\,ICG-Weight\,of\,ICG\,in\,the\,supernatant}{Total\,weight\,of\,nanoparticles}$$

### The photothermal properties of TPNs/ICG-cRGD

The photothermal properties of nanoparticles were measured in vitro. A series of TPNs/ICG-cRGD with different concentrations were exposed to a near-infrared laser (808 nm, 2 w·cm^− 2^) for 5 min. The initial temperature is maintained at around 22 ℃. The temperature changes were recorded by a thermocouple, and photothermal images were obtained by an infrared imager.

### Thrombus targeting assay

#### Platelet and red blood cell extraction

Healthy SD rats were used as the source of blood for platelet and red blood cell isolation. The rat blood was drawn from their veins and collected in 4% citric acid to prevent clotting under anesthesia. The collected blood was centrifuged at 800 rpm for 8 min to separate the platelet-rich plasma. The resulting plasma was further diluted with 5 mL of PBS solution and centrifuged at 3500 rpm for 10 min to obtain the platelets. The platelets were then washed three times with PBS solution. To isolate red blood cells, the whole blood was centrifuged at 2000 rpm and washed with PBS three times.

#### Binding of TPNs/ICG-cRGD to resting platelets and activated platelets in vitro

The specific interaction between TPNs/ICG-cRGD and activated platelets was confirmed by flow cytometry [23]. First, platelet rich plasma was divided into two groups: one group was stimulated with adenosine diphosphate (ADP) at a concentration of 20 µM and incubated at 37 ℃ for 10 min to obtain activated platelets. The other group was treated with an equivalent volume of PBS buffer. Then, both groups of platelets were diluted to a concentration of 2 × 10^6^ platelet·mL^− 1^ with PBS. The TPNs, TPNs/ICG-PEG, TPNs/ICG-cRGD labeled with FITC was incubated at 37℃ for 1 h. Finally, the platelets were washed with cold PBS to remove unbound nanoparticles and analyzed by flow cytometry.

### Thrombolysis of TPNs/ICG-cRGD to platelets poor, platelets rich and whole thrombus in vitro

Platelet poor, platelet rich and whole blood thrombi were prepared according to the reference [24]. Then, various concentrations of TPNs/ICG-cRGD were added to the clots and incubated at 37 °C for 10 min. After that, the thrombi were irradiated with laser (808 nm, 2 w·cm^− 2^). The amount of fibrin and hemoglobin in the supernatant of thrombi was recorded by a UV-vis spectrophotometer at 450 nm and 540 nm, respectively.

### Cytotoxicity study

The RAW264.7, HUVEC, MOVAS cells were seeded in 96-well plates at a density of 5 × 10^3^ cells per well and incubated overnight. Then, various concentrations of TPNs/ICG-cRGD (0–100 µg·mL^− 1^) were added to the wells and incubated for 24 h. After that, the culture medium was replaced with 100 µL MTT solution (0.5 mg·mL^− 1^) and incubated for an additional 4 h. The formazan was dissolved by adding DMSO, and the absorbance was measured at 570 nm using microplate reader (Infinite M200 PRO, TECAN, Austria).

### The antioxidant activity of TPNs/ICG-cRGD in vitro

#### DPPH free radical scavenging activity

Different concentrations of TPNs/ICG-cRGD and acetate buffer (180 µL, 100 mM, pH 5.5) were added to DPPH ethanol solution (0.3 mM, 200 µL), vortexed vigorously in the dark. UV spectra of 400–800 nm were measured after 40 min. A standard curve was prepared to quantify DPPH using UV–vis absorbance at 517 nm, and the DPPH scavenging percentage was calculated in the presence of various concentrations of TPNs/ICG-cRGD.

#### ABTS^+•^ scavenging activity

ABTS^+•^ was produced by mixing 7.4 mM ABTS with 2.6 mM (NH_4_)_2_S_2_O_8_ (1:1, v/v) and then stored in the dark at 4 °C overnight. The ABTS^+•^ solution was diluted with PBS (10 mM, pH 7.4) to an absorbance at 405 nm to get an ABTS^+•^ work solution. Then, 800 µL of the ABTS^+•^ working solution was added to 200 µL of various concentrations of TPNs/ICG-cRGD, Similarly, the percentage of ABTS^+•^ scavenging was calculated based on a standard curve for ABTS^+•^.

#### OH scavenging activity

H_2_O_2_ (0.03%) was used as the standard, the specific operation process was performed according to the instructions of the kit. The effects of different concentrations of nanoparticles on ·OH were calculated based on the absorbance values at 550 nm.

#### O_2_^−^ scavenging activity

Vitamin C (0.15 mg·mL^− 1^) was used as the standard, and the specific operation process was followed according to the instructions of the kit. The effects of different concentrations of nanoparticles on ·O_2_^−^ were calculated based on the absorbance values at 550 nm.

#### NO scavenging activity

**·**NO was produced by NaNO_2_ and determined by a nitric oxide assay kit. Briefly, 50 µL of TPNs/ICG-cRGD were mixed with NaNO_2_ (5 mM) in 100 µL of PBS solution (0.2 M, pH 7.4) and shaken at room temperature. Then, 50 µL of Griess reagent R1 and R2 were added to the mixture and incubated for an additional 0.5 h, The concentration of **·**NO was determined by UV-vis spectra at 550 nm.

### Intracellular RONS scavenging

The RAW264.7 cells (5 × 10^4^ cells per well) were seeded in 24-well plates and incubated overnight. Cells were stimulated by LPS (10 µg·mL^− 1^) for 24 h and then treated with TPNs/ICG-cRGD (70 µg·mL^− 1^) for 24 h. Subsequently, intracellular RONS levels were measured by fluorescent microscopy (ECLIPSE TiS, Nikon, Japan) upon addition of redox-sensitive dye oxidative stress probe (the ROS-ID hypoxia/oxidative stress detection kit with 1:1500 dilution for general RONS), 20 µM 2′,7′-dichlorofluorescin diacetate (DCFH-DA for H_2_O_2_), 5 µM dihydroethidium (DHE for ·O_2_^–^), 10 µM hydroxyphenyl fluorescein (HPF, for ·OH/ONOO^–^), or 5 µM 4-amino-5-methylamino-2′,7′-difluorofluorescein diacetate (DAF-FM DA for ·NO). To quantify the ROS level, the cells were seeded in 6-well plates at a density of 3 × 10^5^ cells per well, and the same treatments were performed as described above. The cells were harvested, and the fluorescence intensity was measured by flow cytometry.

### Thrombolysis of TPNs/ICG-cRGD in vivo

SD rats (100–130 g) were anesthetized by intraperitoneal injection of amobarbital (3%, 400 µL/100 g). The animal experimental model was conducted in accordance with previous studies [10]. In brief, the rat fur on the neck was shaved, and the rat was secured to the operating table with a rope. The carotid artery was exposed by making a midline incision between the chin and sternum and carefully separating the surrounding muscles with fine tweezers. A small piece of plastic wrap was placed under the blood vessel to protect the surrounding tissue, and a 10% FeCl_3_-saturated filter paper (10 × 10 mm) was wrapped around the right carotid artery of the rat for 10 min to induce thrombus formation. Finally, the residual FeCl_3_ was washed with normal saline three times.

The SD rats were randomly allocated into the following groups: control group, sham model group, model group, free ICG + NIR group, TPNs/ICG-PEG + NIR group and TPNs/ICG-cRGD + NIR group. The rats were administered with ICG at a dose of 0.315 mg·kg^− 1^. After 40 min of administration, the neck of the rats was exposed to laser (808 nm, 2w·cm^− 2^) for 15 min. The blood flow of rats was monitored by blood flow imaging perfusion system (PeriCam PSI, Sweden). On the following day, the rats were sacrificed, and their carotid arteries were removed. The thrombus vessels and major organs, including the heart, liver, spleen, lung, and kidney, were washed with PBS and fixed with 4% paraformaldehyde. Hematoxylin-eosin (HE) staining was performed to assess the therapeutic effect of thrombus and pathological changes.

### Serum cytokine determination

The serum was separated from the collected blood by centrifugation. The levels of cytokines including IL-6, IL-8, CRP and TNF-α in the serum were determined by ELISA kits according to the manufacturer’s instructions.

### Safety evaluation

#### Hemolysis evaluation

The collected red blood cells were diluted to one-tenth of their volume with PBS. The 200µL TPNs/ICG-cRGD with different concentrations were added into 1.3 mL freshly isolated blood and incubated at 37℃ for 4 h. Next, the solution was centrifuged at 3000 rpm for 10 min, and the supernatant was collected and measured for absorbance at 540 nm. PBS was used as the negative control, while deionized water was used as the positive control. Hemolysis ratio was calculated by the following formula:

$$hemolysis\%=$$

$$\frac{sample\,absorbance-negative\,control\,absorbance}{positive\,control\,absorbance-negative\,control\,absorbance}$$

$$\times 100\%$$

#### Blood routine and four items of blood coagulation test

Before testing the samples, we did perform specific processing steps. For the blood routine test, fresh rat blood was added to tubes containing the anticoagulant EDTA-K2. The mixture was gently inverted to ensure thorough mixing and then placed on ice for testing. Subsequently, the samples were tested by an automatic blood cell analyzer (BC-2800vet, Mindray, China). As for the four items of the blood coagulation test, fresh rat blood was added to tubes containing sodium citrate as the anticoagulant. After inverting the tubes for mixing, the samples were centrifuged at 4 °C and 1000×g for 10 min. The upper layer of pale yellow was analyzed by an automatic blood coagulation analyzer (RAC-1830, Rayto, China).

### Pharmacokinetics

In this study, intravenous doses of ICG (0.35 mg·kg^− 1^), TPNs/ICG-PEG (7.8 mg·kg^− 1^) and TPNs/ICG-cRGD (7.8 mg·kg^− 1^) were administered in SD rats. Post-administration, Blood samples were collected at 2, 5, 10, 15, 30, 60, 120, 240, 360, 1440, and 2880 min. The collected blood was centrifuged at 1000 ×g at 4 ℃ for 10 min to obtain plasma. The plasma samples were then analyzed by high-performance liquid chromatography with tandem mass spectroscopy (AB, USA).

### In vivo imaging and biodistribution

To investigate the biodistribution of TPNs/ICG-cRGD and ICG in vivo, TPNs/ICG-cRGD (7.8 mg/kg^− 1^) and ICG (0.35 mg/kg^− 1^) were injected into SD rats. The biodistribution was monitored by capturing images at different time point (5, 10, 20, 30, 40 and 50 min) using in vivo imaging system (PerkinElmer, USA). 48 h post-injection, the rats were euthanized, and major organs, including the heart, liver, spleen, lung, kidney were collected for fluorescence imaging.

### Statistical analysis

All the quantitative results were reported as mean ± standard deviation (SD) and presented as graphs by Prism software version 8 (GraphPad Software, San Diego, CA, USA). Student’s T-test and one-way analysis of variance (ANOVA) were used to assess the differences. The level of significance was set at *p < 0.05, **p < 0.01, ***p < 0.001, ****p < 0.0001.

## Results and discussion

### Preparation and characterizations of TPNs/ICG-cRGD

**Fig. 1:**
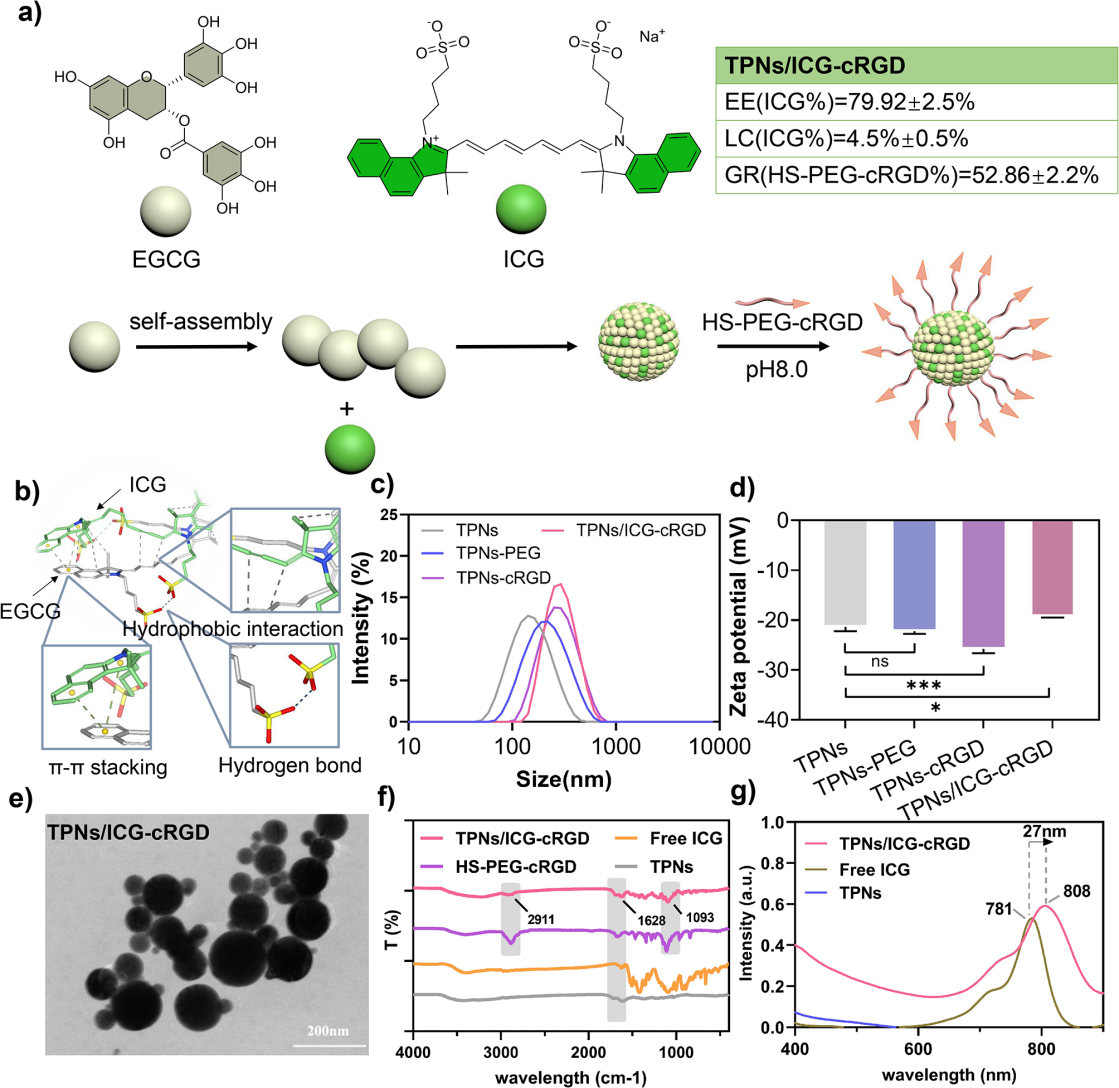
**a** Schematic representation of the TPNs/ICG-cRGD construction. **b** Computational docking simulation results depicting EGCG and ICG interactions in an aqueous medium. **c** Size distribution and **d** ζ potential of TPNs, TPNs-PEG, TPNs-cRGD, and TPNs/ICG-cRGD. **e** TEM images of TPNs/ICG-cRGD with a scale bar of 200 nm. **f** FTIR spectrum of TPNs/ICG-cRGD, Free ICG, TPNs, and HS-PEG-cRGD. **g** Ultraviolet spectrum of TPNs/ICG-cRGD, Free ICG, and TPNs. EE (encapsulation efficiency), LR (drug loading rate), GR (graft rate)

The ICG-loaded TPNs were meticulously prepared through the pre-mixing of EGCG and ICG, which facilitated their intermolecular interactions. Molecular simulations revealed several significant forces at play within the EGCG/ICG system, including hydrophobic interactions, hydrogen bonds, and π-π stacking (Fig. 1b). The introduction of Mn^2+^ triggered a rapid oxidation and polymerization of EGCG, leading to its assembly into TPNs, concurrent with the encapsulation of ICG (Fig. 1a). Subsequently, PEG-cRGD, bearing a terminal thiol group, was conjugated to the nanoparticle surface via a nucleophilic addition reaction. This process, characterized by its simplicity and single-step nano-precipitation, achieved a high ICG encapsulation efficiency, approximately 80% (Figure S1), and a PEG-cRGD conjugation ratio of approximately 53% (Figure S2). For comparison, control nanoparticles, namely TPNs-PEG and TPNs-cRGD, were also prepared.

Following surface modifications with PEG or PEG-cRGD, there was a slight increase in the hydrodynamic size of the nanoparticles (Fig. 1c), while all nanoparticles maintained a negative charge (Fig. 1d). Notably, TPNs/ICG-cRGD demonstrated minimal variations in particle size when exposed to various aqueous media for up to 48 h (Figure S3), underscoring their high colloidal stability for potential biological applications. It is worth mentioning that the nanoparticles exhibited a notably larger colloidal size in media containing fetal bovine serum (FBS) compared to buffered solutions, likely due to the formation of a surface protein corona. Transmission Electron Microscopy (TEM) images provided insight into the morphological characteristics of these nanoparticles. TPNs-cRGD exhibited a spherical structure with a consistent size of around 60 nm (Figure S4a). In contrast, the size of TPNs/ICG-cRGD increased significantly due to the co-assembly of ICG (Fig. 1e and Figure S4b).

To validate the structural changes, Fourier Transform Infrared Spectroscopy (FTIR) was conducted (Fig. 1f). In comparison to TPNs and free ICG, TPNs/ICG-cRGD exhibited a new peak at 2911 and 1093 cm^− 1^, which corresponded to the vibrational peak of HS-PEG-cRGD, indicating successful conjugation of TPNs with HS-PEG-cRGD. The structural changes were further confirmed through UV-Vis spectroscopy (Fig. 1g), where the characteristic peak of ICG was also observed in TPNs/ICG-cRGD with a slight redshift of approximately 27 nm. This shift was attributed to the non-covalent binding between ICG and EGCG, which serves to reduce the dimerization of ICG. Collectively, these characterization data establish that EGCG and ICG effectively formed nanoparticles through self-assembly, and HS-PEG-cRGD was successfully coupled to TPNs.

### Photothermal capability of TPNs/ICG-cRGD

**Fig. 2:**
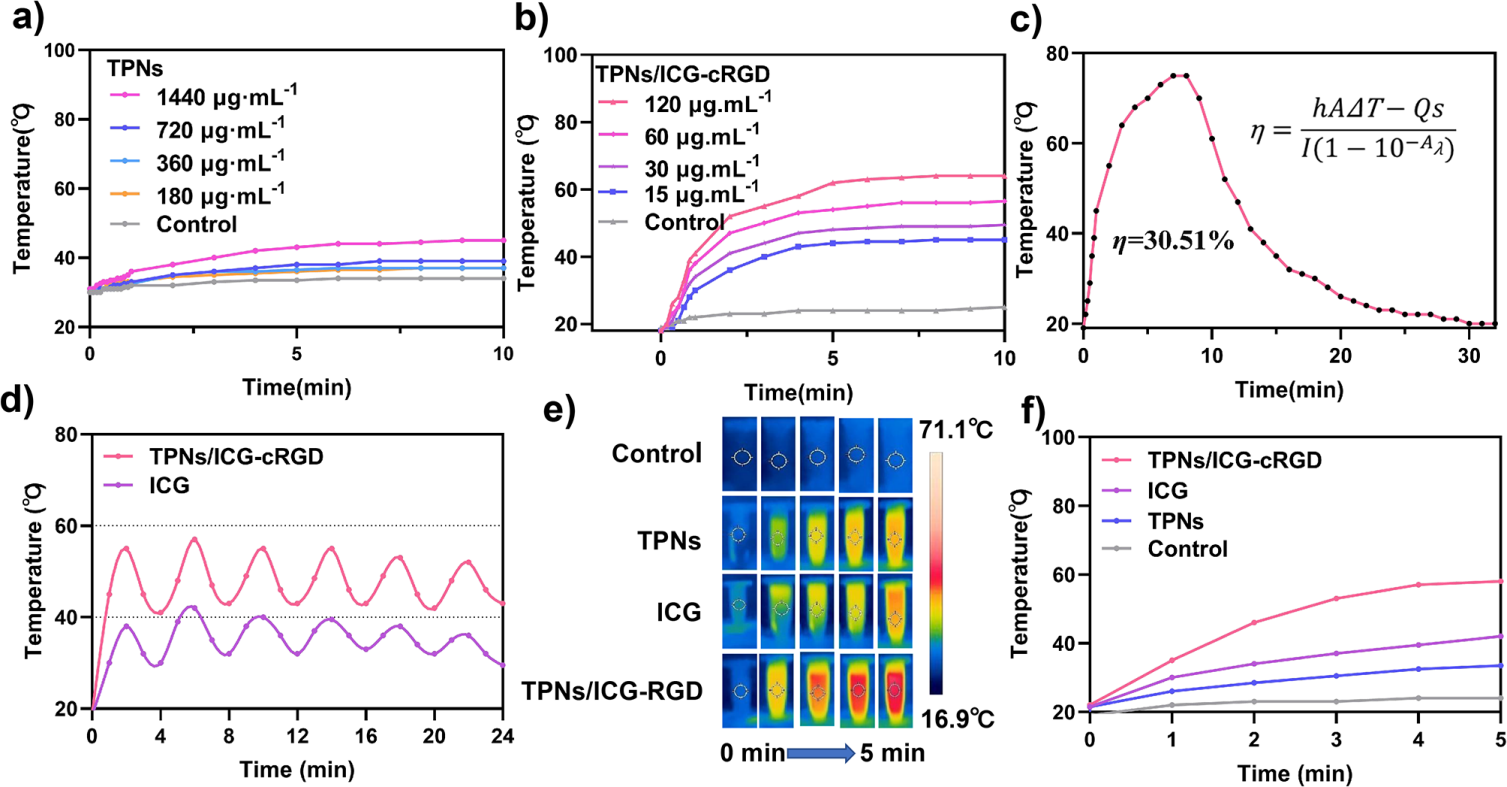
**a** Temperature elevation of TPNs at varying concentrations under 808 nm laser irradiation (2 W·cm^− 2^, 10 min). **b** Temperature elevation of TPNs/ICG-cRGD at different concentrations under 808 nm laser irradiation (2 W·cm^− 2^, 10 min). **c** Photothermal conversion of TPNs/ICG-cRGD under 808 nm laser irradiation (2 W·cm^− 2^). **d** Temperature changes in TPNs/ICG-cRGD and free ICG over six cycles of repeated laser irradiation. **e** Infrared thermal images of TPNs/ICG-cRGD, TPNs and free ICG under 808 nm laser irradiation (2 W·cm^− 2^, 5 min). **f** Peak temperature profiles of TPNs/ICG-cRGD, TPNs and free ICG under 808 nm laser irradiation (2 W·cm^− 2^, 5 min)

In line with the photothermal properties observed in other self-polymerized nanoparticles [25, 26], TPNs exhibited a photothermal effect (Fig. 2a). However, the overall photothermal conversion efficiency was relatively low, necessitating a concentration exceeding 1 mg·mL^− 1^ to achieve a substantial effect. With the incorporation of ICG, a notable enhancement in the photothermal effect was observed, with a concentration-dependent trend (Fig. 2b). To quantitatively assess the photothermal conversion efficiency (η), a 10-minute irradiation was administered, followed by a natural cooling process until the original temperature was regained. Utilizing the equation presented in Fig. 2c, the calculated η was determined to be 30.51%.

To evaluate the photostability of TPNs/ICG-cRGD in comparison to free ICG molecules, solutions of TPNs/ICG-cRGD and free ICG were subjected to NIR laser irradiation for 2 min, followed by a 2-minute cooling period without NIR laser irradiation. The results, as illustrated in Fig. 2d, demonstrated that TPNs/ICG-cRGD exhibited temperature elevations of 45 °C, 57 °C, 55 °C, 55 °C, 53 °C, and 52 °C over six cycles, while the free ICG solution reached temperature elevations of 38 °C, 42 °C, 40 °C, 39.5 °C, 38 °C, and 36 °C, respectively. Subsequently, the temperature changes of these formulations under continuous laser irradiation were assessed (Fig. 2e and f). A notably faster temperature increase was observed for TPNs/ICG-cRGD in comparison to free ICG and TPNs. Consequently, both the photostability and photothermal capability of ICG were significantly enhanced following its encapsulation within nanoparticles. In addition, an obvious synergistic effect was observed between ICG and TPNs for photothermal conversion, demonstrating the benefit of their integration.

### Thrombus targetability of TPNs/ICG-cRGD

**Fig. 3:**
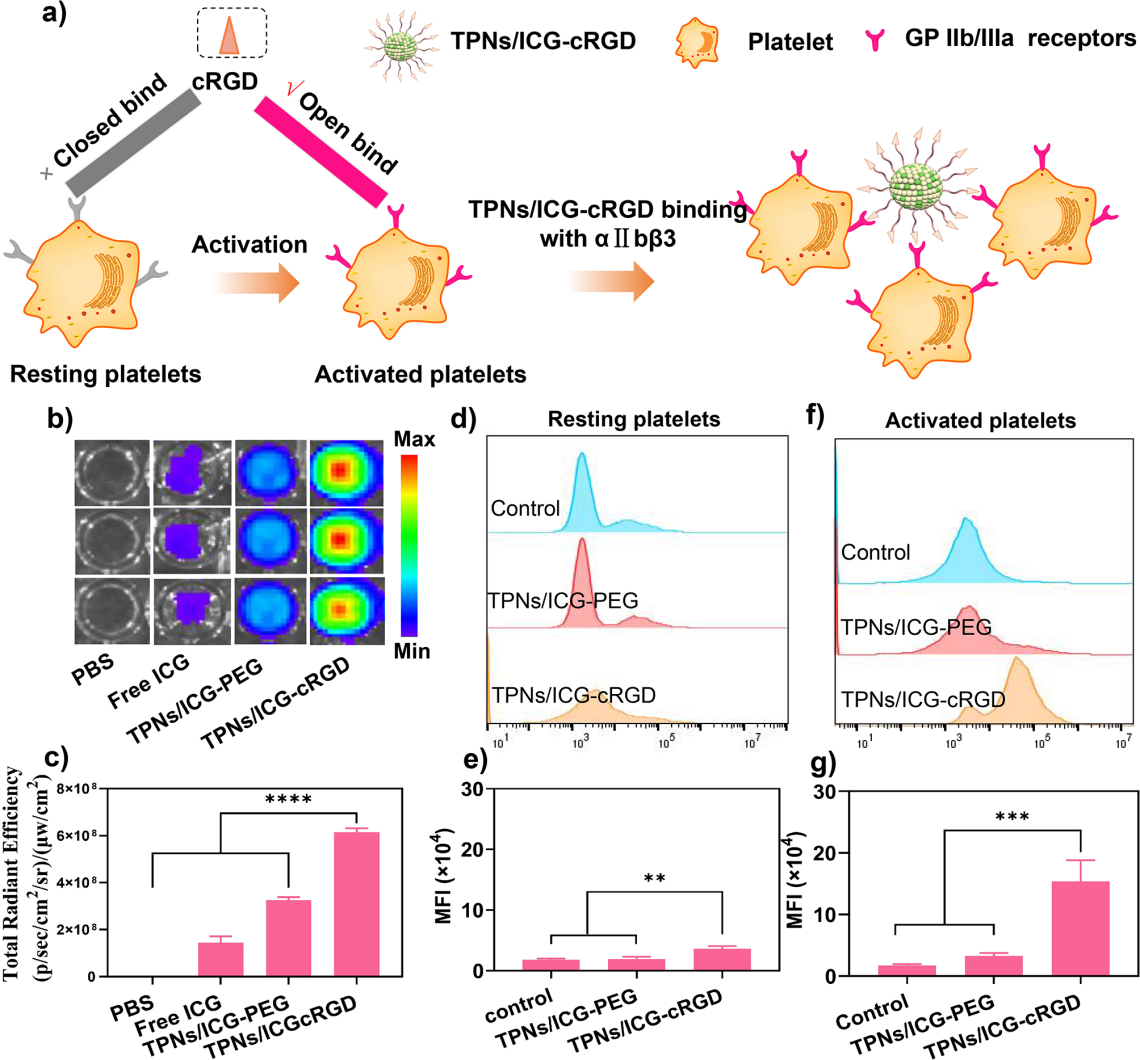
**a** Schematic representation illustrating the targeted binding of TPNs/ICG-cRGD to activated platelets. **b** Fluorescence signals observed in artificial blood clots following incubation with various treatments. **c** Quantitative analysis of fluorescence intensity within artificial blood clots. **d** Flow cytometry analysis of the fluorescence in resting platelets with various treatments. **e** Mean fluorescence intensity in resting platelets. **f** Flow cytometry analysis of the fluorescence in activated platelets. **g** Mean fluorescence intensity in activated platelets (*n* = 3, mean ± SD)

In pursuit of thrombus-specific delivery, the surface modification of nanoparticles with cRGD was employed to facilitate binding with activated platelets within thrombi. In the quiescent state, integrin αIIbβ3 retains a low-affinity conformation; however, upon activation, it transitions to a high-affinity state for cRGD ligands, as illustrated in Fig. 3a [27, 28]. This transition allows for the targeted delivery of nanoparticles to thrombi [24]. To assess whether the presence of cRGD could enhance the selectivity of TPNs/ICG-cRGD in binding to activated platelets, an artificial blood clot model was created for evaluation.

As shown in Fig. 3b and c, TPNs/ICG-cRGD exhibited significantly stronger fluorescence signals within the clots compared to free ICG and TPNs/ICG-PEG, underscoring the pivotal role of cRGD in thrombus targeting. Building upon these promising results, we further scrutinized the binding behavior of these nanoparticles by employing Flow cytometry analysis, with the nanoparticles labeled using fluorescein isothiocyanate (FITC). As depicted in Fig. 3d and e, both TPNs/ICG-PEG and TPNs/ICG-cRGD displayed minimal fluorescence in resting platelets. In contrast, for activated platelets, TPNs/ICG-cRGD exhibited a specific increase in fluorescence (Fig. 3f and g). Quantitative analysis of the fluorescence intensity indicated that the selective binding of TPNs/ICG-cRGD to activated platelets was approximately five times higher compared to TPNs/ICG-PEG (Fig. 3g). These findings provide robust evidence that the presence of cRGD on the nanoparticle surface significantly enhances specific targeting to activated platelets within thrombi, establishing a solid foundation for subsequent thrombolytic therapy.

### Photothermal thrombolysis of TPNs/ICG-cRGD

**Fig. 4:**
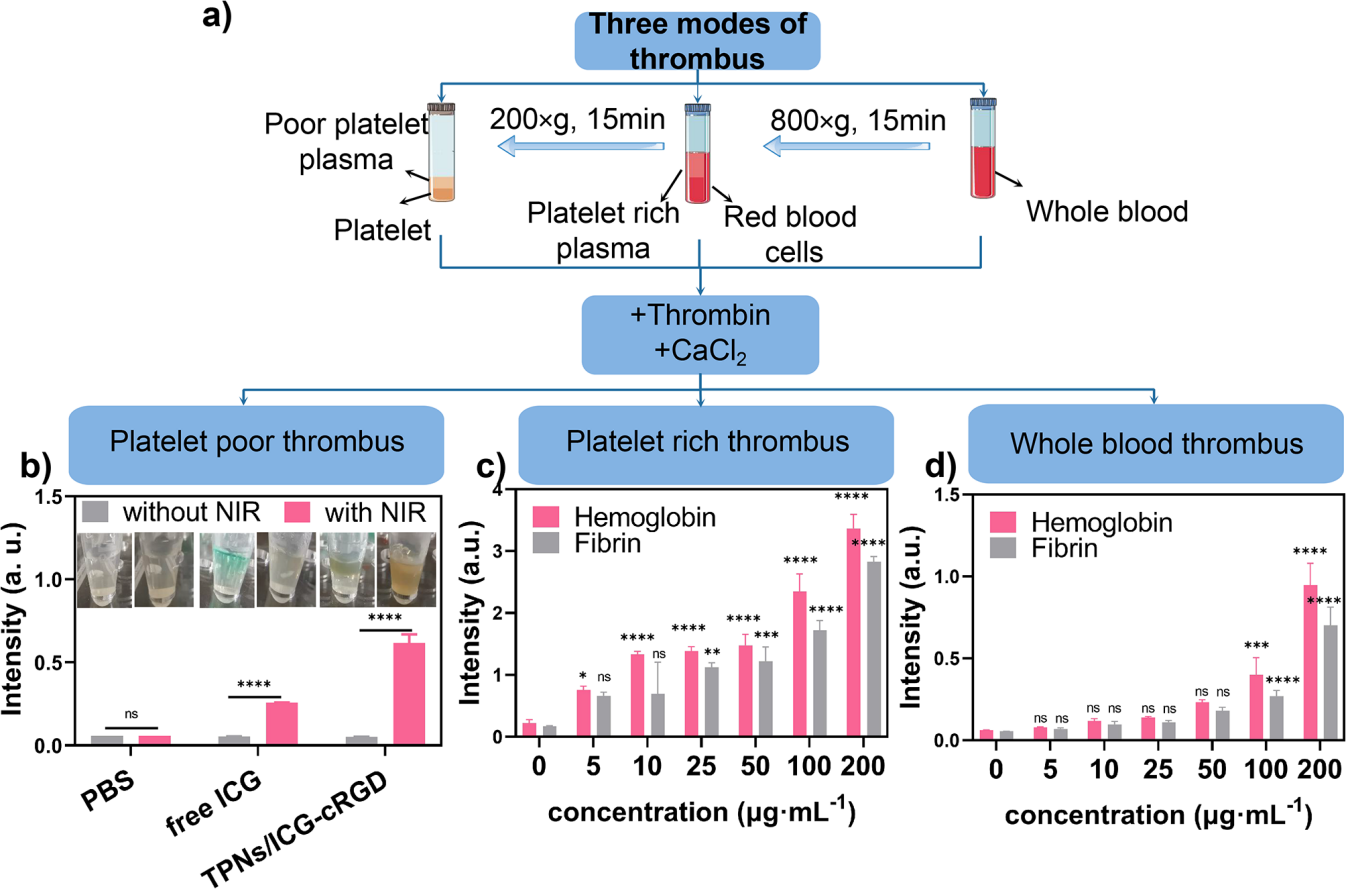
**a** Preparation of platelet-poor, platelet-rich, and whole blood thrombus. **b** UV absorption (450 nm) of fibrin dissolved in the supernatants following treatments (*n* = 3). **c** UV absorption (450 nm) of fibrin and UV absorption (540 nm) of hemoglobin after treatment with varying concentrations of TPNs/ICG-cRGD in platelet rich thrombus. **d** UV absorption (450 nm) of fibrin and UV absorption (540 nm) of hemoglobin after treatment with varying concentrations of TPNs/ICG-cRGD in whole blood thrombus

Motivated by the notable photothermal conversion efficiency and thrombus-targeting capabilities of TPNs/ICG-cRGD, we proceeded to investigate the thrombolysis activity of these nanoparticles in vitro. Three distinct thrombus models, namely whole blood thrombus, platelet-rich thrombus, and platelet-poor thrombus, were prepared for evaluation (Fig. 4a). In the absence of any treatment, the platelet-poor thrombus at the bottom of the tubes remained intact even after multiple tube inversions. In contrast, blood clots were effectively disrupted after thrombolytic treatment with free ICG and TPNs/ICG-cRGD. Under the same conditions, the fibrin within the clot was dispersed into the surrounding medium (Fig. 4b). The dissolved fibrin in the supernatants was quantitatively determined using UV-Vis spectroscopy. The thrombolytic efficiency of free ICG and TPNs/ICG-cRGD was approximately 4 times and 11 times greater than that of the phosphate-buffered saline (PBS) control when subjected to laser irradiation (Fig. 4b). This observation underscores the effectiveness of photothermal thrombolysis.

Given the conspicuous thrombolytic effect of TPNs/ICG-cRGD on platelet-poor thrombus, we proceeded to explore the impact of TPNs/ICG-cRGD on whole blood thrombus and platelet-rich thrombus. The absorbance of the supernatant was assessed at 450 nm and 540 nm, serving as indicators for fibrin and hemoglobin, respectively. Importantly, the thrombolytic effect of the nanoparticles was found to be consistent across all thrombus types (Fig. 4c and d). Moreover, the thrombolytic effect on both whole blood thrombus and platelet-rich thrombus was dose-dependent over the concentration range to 200 µg·mL^− 1^. In summary, the in vitro results demonstrate the positive effects of TPNs/ICG-cRGD on all three modes of thrombus.

### RONS scavenging activities of TPNs/ICG-cRGD

When thrombosis occurs, it leads to tissue damage, triggering the release of various pro-inflammatory factors that stimulate the production of reactive oxygen and nitrogen species (RONS) by different cell types, including immune cells and endothelial cells [29]. The presence of RONS during thrombus formation can significantly impact the inflammatory response, endothelial function, clot growth, and subsequent complications [30]. Therefore, managing RONS levels is essential in preventing further clot formation and promoting effective healing [16]. Fortunately, our previous work demonstrated that TPNs possess inherent antioxidative properties capable of scavenging a broad spectrum of RONS [18], which is advantageous for anti-thrombus therapy. To validate their efficacy, we initially assessed the capability of TPNs/ICG-cRGD in a test tube, using physiologically relevant RONS including hydroxyl radical (·OH), superoxide anion radical (·O_2_^–^), and nitric oxide radical (NO·), generated through the Fenton reaction, xanthine and xanthine oxidase reaction, and sodium nitroprusside, respectively. Each type of RONS was visually identifiable through a distinct pink color. Significantly, this color faded progressively with increasing concentrations of TPNs/ICG-cRGD treatment, reaffirming the RONS scavenging activities of TPNs were maintained after ICG loading and surface cRGD modification. For quantitative assessment, UV-Vis spectra indicative of different RONS were measured (Figure S5), revealing a concentration-dependent decrease in absorbance for all RONS species following incubation with TPNs/ICG-cRGD. Specifically, the scavenging rates for ·OH, ·O_2_^–^, and NO· at a concentration of 100 µg·mL^− 1^ TPNs/ICG-cRGD were calculated to be 73.7%, 43.9%, and 15%, respectively.

**Fig. 5:**
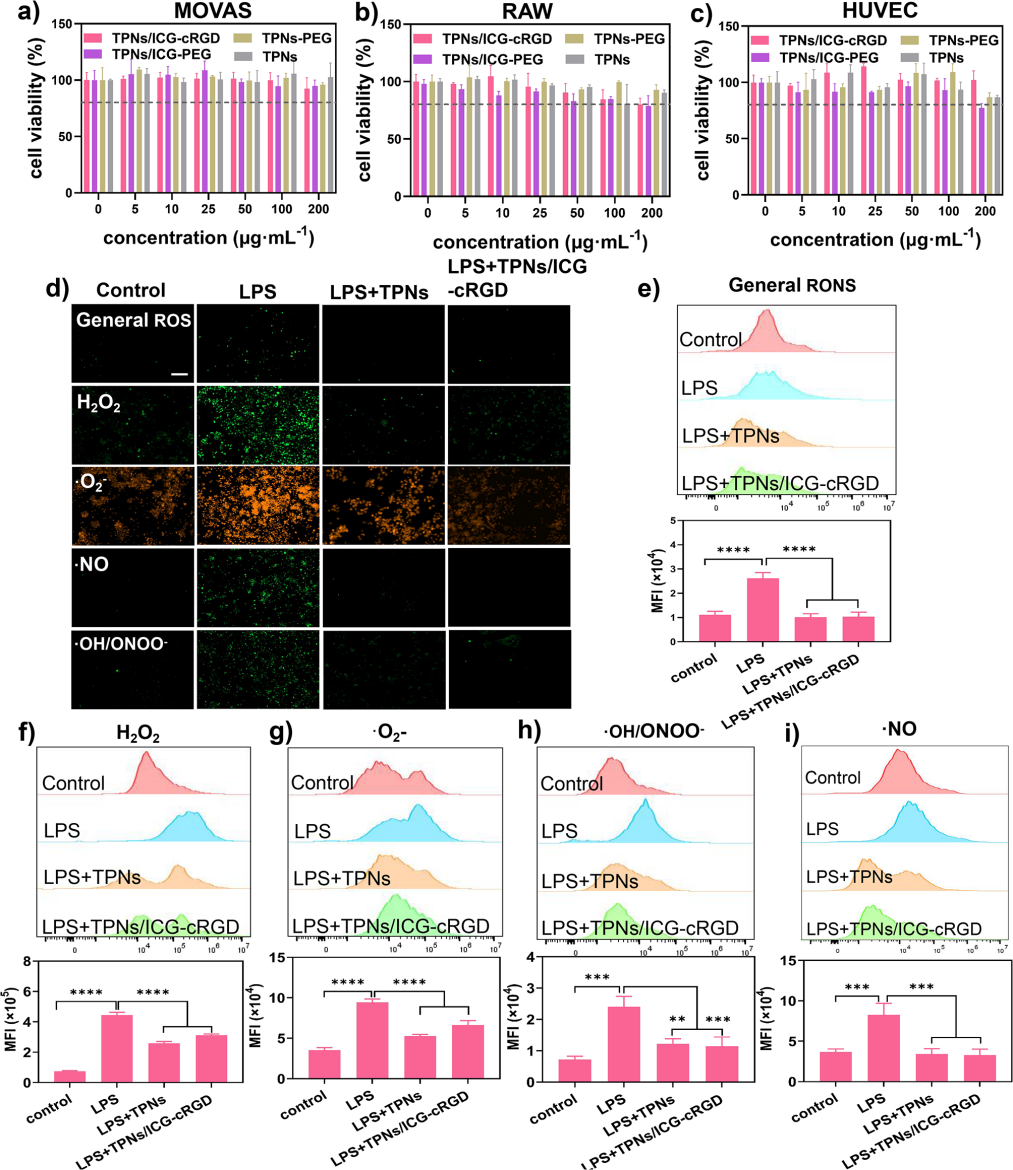
Cell viability of (**a)** MOVAS, (**b)** RAW 264.7, and (**c)** HUVEC upon exposure to varying concentrations of TPNs, TPNs-PEG, TPNs/ICG-PEG, and TPNs/ICG-cRGD. (**d)** Fluorescence micrographs, (**e-i)** flow cytometry data and quantification of intensity illustrating the ability of TPNs/ICG-cRGD to scavenge different types of RONS in RAW264.7 cells with LPS pretreatment (*n* = 3). Scale bar = 100 μm

Given the promising RONS scavenging effects observed in a test tube, we proceeded to investigate these effects within cells. To do so, we first examined the cell viability of TPNs/ICG-cRGD. As macrophages, endothelial cells, and smooth muscle cells are known to play a significant role in thrombosis [31–33], we evaluated the cell viability of TPNs, TPNs-PEG, TPNs/ICG-PEG, and TPNs/ICG-cRGD in these cell lines. Remarkably, these nanoparticles exhibited minimal cytotoxicity across a wide concentration range (up to 100 µg·mL^− 1^) (Fig. 5a-c), reflecting their high biocompatibility. To induce oxidative stress within the cells, we stimulated them with lipopolysaccharide (LPS), leading to an excessive production of RONS as indicated by the intensified fluorescence of the oxidative stress probe ROS-ID (Fig. 5d), signifying elevated levels of total RONS. Following incubation with TPNs and TPNs/ICG-cRGD, the fluorescence signal notably diminished, illustrating the general RONS scavenging capabilities. We further quantified this effect using flow cytometry, which demonstrated that both TPNs and TPNs/ICG-cRGD could reduce the fluorescence to nearly background levels (Fig. 5d), affirming its robust efficacy. We also assessed the activity of TPNs/ICG-cRGD in clearing different types of radicals within cells, including reactive oxygen species (ROS) of hydrogen peroxide (H_2_O_2_) and superoxide anion radical (·O_2_^–^), as well as reactive nitrogen species (RNS) of nitric oxide (·NO) and peroxynitrite (ONOO–). These species were detected using fluorescent indicators, with results showing a significant reduction in ROS and RNS within cells following treatment with TPNs/ICG-cRGD, demonstrating their versatile capacity to scavenge various types of RONS both in solution and within cells, thereby protecting cells from oxidative damage.

### In vivo antithrombotic therapy with TPNs/ICG-cRGD

Having established the favorable characteristics of TPNs/ICG-cRGD in vitro, it was imperative to proceed with in vivo investigations through tail vein injection. Prior to intravenous injection, the hemocompatibility of the nanoparticles was assessed using red blood cells. Over a concentration range of 0–100 µg/mL, TPNs/ICG-cRGD exhibited less than 5% hemolysis (Figure S6), confirming their suitability for intravenous administration. To study the pharmacokinetics, the blood samples were dynamically collected, and the ICG concentration was measured via LC-MS/MS. Compared to that of free ICG, the half-life of TPNs/ICG-PEG and TPNs/ICG-cRGD significantly increased by 7- and 5.7-fold (Figure S7a, b), demonstrating the long circulation effect of the nanoparticles [34]. Notably, TPNs/ICG-cRGD displayed high signal at thrombotic site during the initial one-hour observation (Figure S8a), demonstrating the targeting effect of the nanoparticles. At 48 h post-injection, the mice were sacrificed, and major organs were collected for ex vivo observation. Both TPNs/ICG-PEG and TPNs/ICG-cRGD showed high fluorescence in liver and kidneys (Figure S8b, c), suggesting the major organs for nanoparticles elimination.

**Fig. 6:**
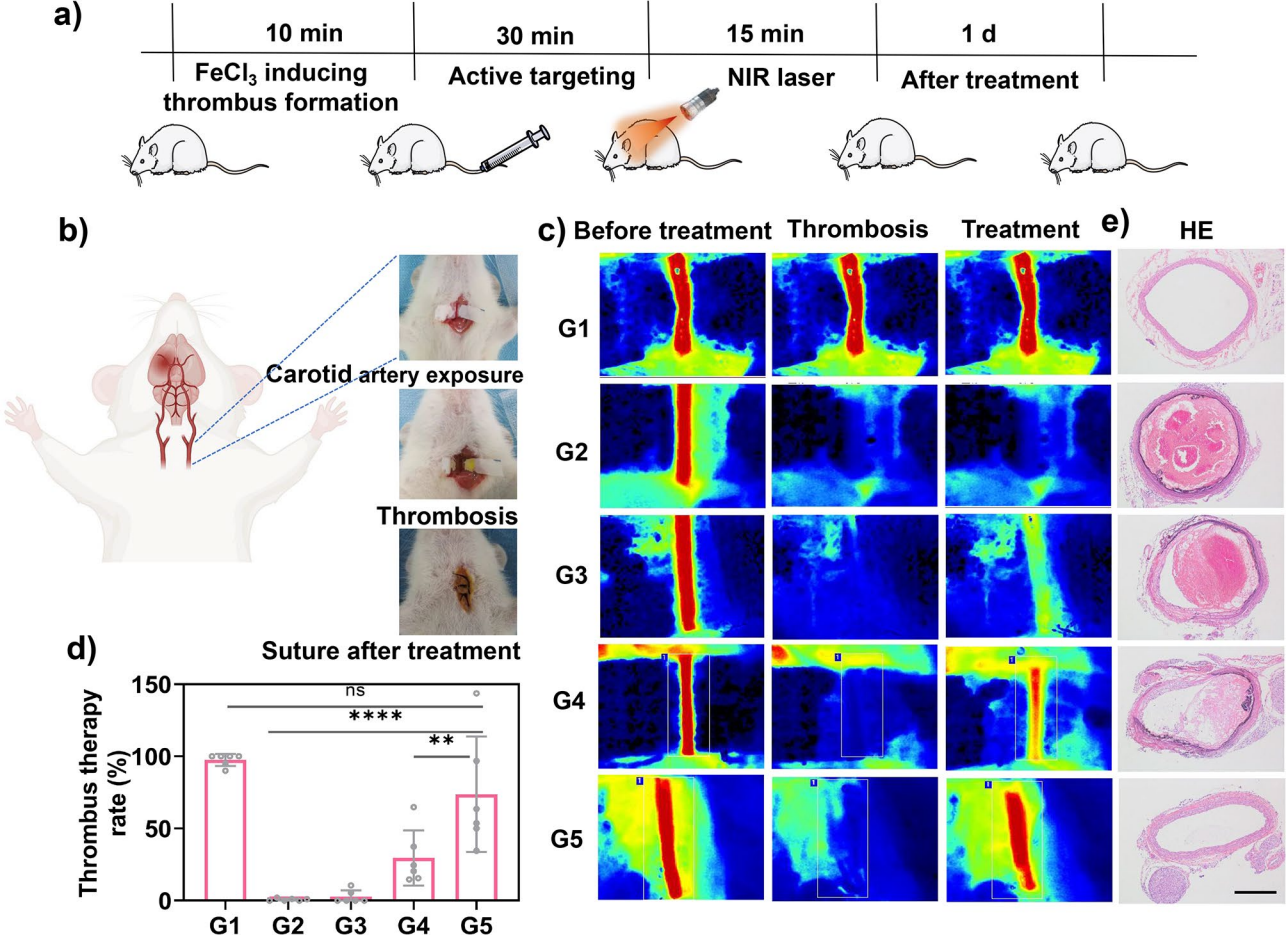
**a** Schematic representation outlining the experimental design for antithrombotic therapy. **b** Schematic depiction of the FeCl_3_-induced rat carotid arterial thrombosis model. **c** Blood flow images and **d** quantitative plot of recanalization rate with different treatment. **e** HE staining images from different treatment groups. Scale bar = 100 μm. G1, Sham model group; G2, Model group; G3, Free ICG (0.35 mg·kg^− 1^) + NIR group; G4, TPNs/ICG-PEG (7.8 mg·kg^− 1^) + NIR group; G5, TPNs/ICG-cRGD (7.8 mg·kg^− 1^) + NIR group

Next, the therapeutic efficacy of the nanoparticles was evaluated. To this end, the experimental framework is delineated in Fig. 6a, featuring an FeCl_3_-induced rat carotid artery thrombosis model (Fig. 6b). Thirty minutes post-injection, laser irradiation was employed to facilitate photothermal thrombolysis, while color Doppler flow imaging was utilized to evaluate thrombolytic efficacy. In the sham model group, no alterations in blood flow were observed, while the model group exhibited persistent blood flow obstruction (Fig. 6c), validating successful thrombus induction. Subsequent treatments with free ICG, TPNs/ICG-PEG, and TPNs/ICG-cRGD led to varying degrees of blood flow recovery, with the most pronounced effect seen in the TPNs/ICG-cRGD group, outperforming both the TPNs/ICG-PEG and free ICG groups. Quantitative analysis further substantiated that the blood flow reperfusion rate was highest following TPNs/ICG-cRGD treatment (Fig. 6d). This superior thrombolytic effect can be attributed to the targeted delivery and enhanced photothermal performance of the nanoparticles, as evidenced by a 28-fold improvement compared to the free ICG group and a 2.5-fold increase compared to the TPNs/ICG-PEG group. Additionally, samples were collected from the carotid artery thrombus site in rats, and histological examination via Hematoxylin and Eosin (HE) staining was performed (Fig. 6e). The carotid artery sections from the model group displayed vessel occlusion, which was mitigated following treatment with various formulations, consistent with the results obtained from color Doppler flow imaging. Once again, TPNs/ICG-cRGD exhibited the most favorable efficacy, underscoring its potential application for targeted thrombolysis.

### In vivo anti-inflammatory effect of TPNs/ICG-cRGD

**Fig. 7:**
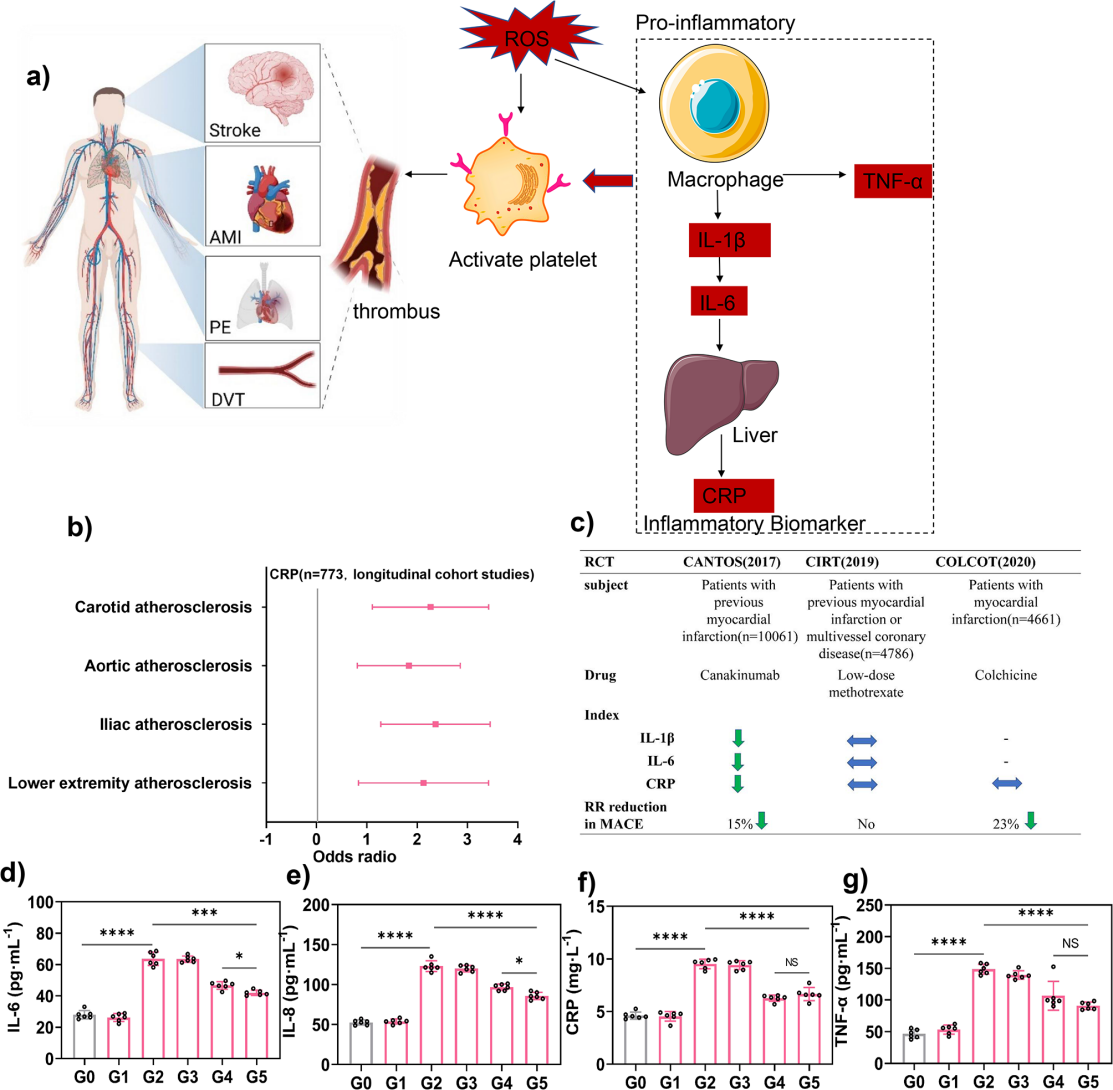
**a** Key cellular and molecular mediators of inflammation in thrombus. **b** Odds ratios (ORs) and 95% confidence intervals (CIs) for the progression of atherosclerosis linked to traditional cardiovascular risk factors and CRP. **c** A comparison of the similarities and differences between the CANTOS, CIRT, and COLCOT clinical trials. **d** IL-6, **e** IL-8, **f** CRP, and **g** TNF-α levels in rats after various treatments. **AMI**, acute myocardial infarction; **PE**, pulmonary embolism; **DVT**, deep venous thrombosis; **RR**, relative risk; **MACE**, major adverse cardiovascular events (composite of cardiovascular death, myocardial infarction, or stroke); **RCT**, randomized controlled trial. The green arrow denotes a reduction, while the blue arrow indicates no change. **G0**, Control group; **G1**, Sham model group; **G2**, Model group; **G3**, Free ICG (0.35 mg/kg) + NIR group; **G4**, TPNs/ICG-PEG (7.8 mg/kg) + NIR group; **G5**, TPNs/ICG-cRGD (7.8 mg/kg) + NIR group. *n* = 6, mean ± SD

Effective anti-inflammatory therapy is pivotal in the context of thrombus treatment. While thrombolysis can restore blood flow by dissolving the thrombus, the local oxidative stress at the thrombus site triggers inflammatory responses, causing endothelial cell damage and the recruitment of inflammatory cells, thereby promoting the formation of new thrombi and recurrence of the disease. For instance, interleukin-6 (IL-6) induces the expression of the C-reactive protein (CRP) gene in the liver, resulting in elevated CRP levels (Fig. 7a). This pro-inflammatory response, coupled with the presence of RONS, sustains platelet activation and thrombus formation [35]. Atherosclerosis creates a conducive environment for thrombotic disease development [36], and a longitudinal cohort study has shown an odds ratio of 2.1 (95% CI, 1.2 to 3.5) for the progression of carotid atherosclerosis associated with elevated CRP levels [37], indicating that CRP levels can serve as a predictor of thrombotic risk (Fig. 7b). In randomized controlled clinical trials, such as the CANTOS trial, strong evidence has been presented that the inhibition of the IL-1β/IL-6 signaling cascade can significantly reduce cardiovascular risk (15%), (Fig. 7c) [38–40]. To summarize, prior basic experiments, prospective cohort studies, and randomized controlled trials have underscored the importance of controlling inflammatory factors in the prevention and prognosis of thrombotic diseases. Nonetheless, there is a scarcity of reports regarding treatment strategies that combine thrombolysis and anti-inflammatory therapy.

Given the robust RONS scavenging capability of TPNs/ICG-cRGD observed in vitro, we postulated that this nanomaterial also possesses anti-inflammatory potential in vivo. To explore this, we assessed the levels of various inflammatory factors, including IL-6, IL-8, CRP, and TNF-α, following treatment (Fig. 7d-g). In comparison to the control group, cytokine levels remained unaltered in the sham group. However, they increased significantly in model mice, indicative of a pronounced inflammatory response following thrombus formation. While free ICG demonstrated substantial antithrombotic activity, it failed to alleviate inflammation, as all cytokines remained at elevated levels. Notably, both TPNs/ICG-PEG and TPNs/ICG-cRGD significantly reduced cytokine levels, substantiating the anti-inflammatory effect of these nanoparticles. In this context, TPNs/ICG-cRGD exhibited superior performance, which can be attributed to its targeted delivery to the thrombus site. In summary, TPNs/ICG-cRGD achieved effective targeted delivery and exerted dual functions in antithrombosis and anti-inflammation, offering a promising approach for concurrent thrombus therapy.

**Fig. 8:**
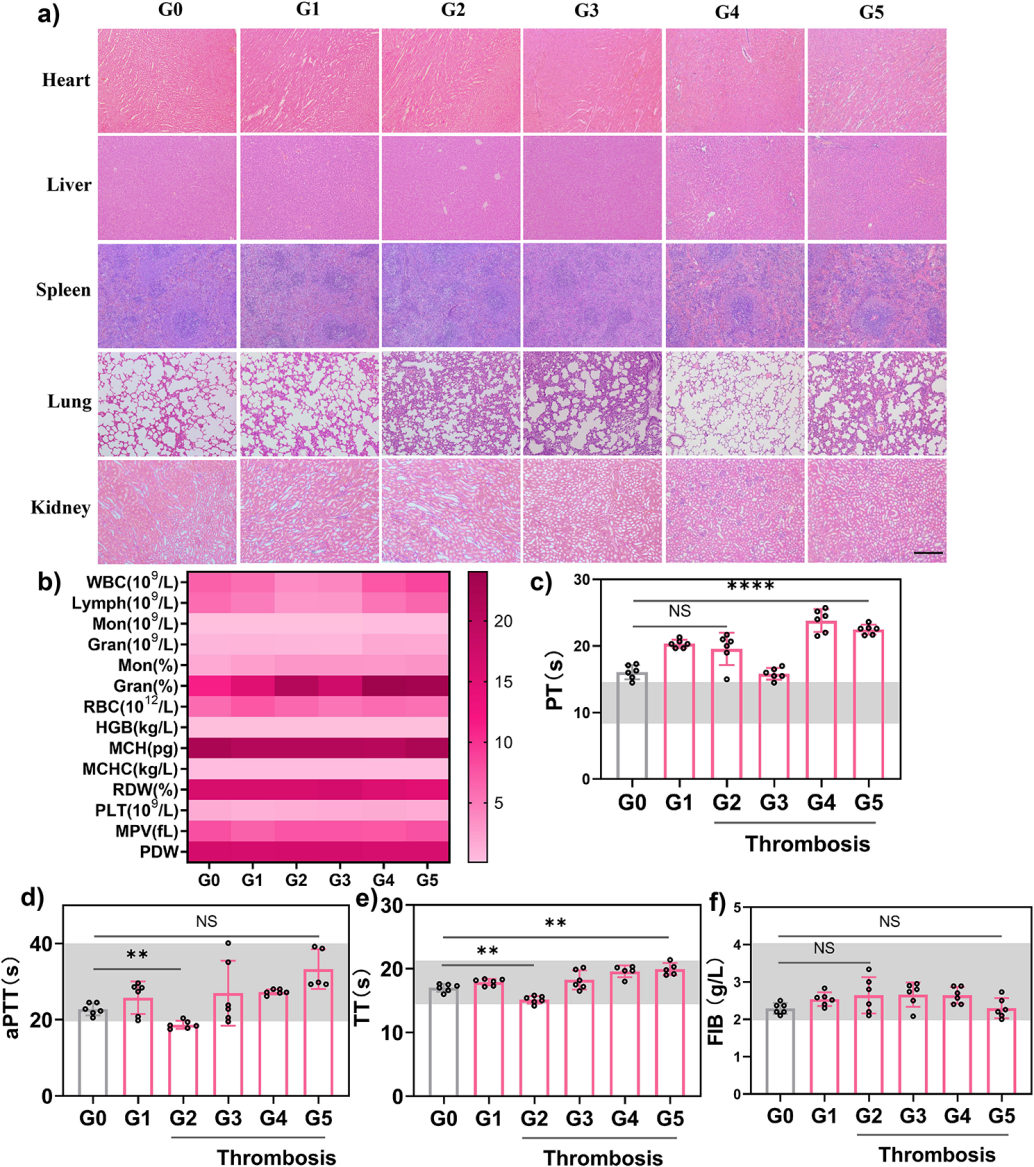
**a** Hematoxylin and eosin (H&E) staining of major organs in healthy rats after various treatments. **b** Blood routine analysis of SD rats after various treatments. **c-f** Blood coagulation parameters (PT, aPTT, TT, FIB) in rat plasma after various treatments. *n* = 6, mean ± SD. Scale bar = 100 μm. **G0**, Control group; **G1**, Sham model group; **G2**, Model group; **G3**, Free ICG (0.35 mg·kg^− 1^) + NIR group; **G4**, TPNs/ICG-PEG (7.8 mg·kg^− 1^) + NIR group; **G5**, TPNs/ICG-cRGD (7.8 mg·kg^− 1^) + NIR group; WBC, white blood cell; Lymph, lymphocyte; Mon, monocyte; Gran, granulocyte; RBC, red blood cell; HGB, hemoglobin; MCH, mean corpuscular hemoglobin; MCHC, mean corpuscular hemoglobin concentration; RDW, red blood cell volume distribution width; PLT, platelet count; MPV, mean platelet volume; PDW, platelet distribution width; PT, prothrombin time; aPTT, activated partial thrombin time; TT, thrombin time; FIB, Fibrinogen

### Safety evaluation

To assess the biosafety of the nanomedicine, comprehensive evaluations were conducted. Hematoxylin and eosin (H&E) staining revealed no pathological changes in major organs after various therapies, indicating the absence of acute toxicity (Fig. 8a). Routine blood analysis was performed for 14 different factors. Only a slight increase in granulocyte percentage was observed for TPNs/ICG-PEG and TPNs/ICG-cRGD, but these results fell within acceptable ranges and did not exhibit significant abnormalities (Fig. 8b). Additionally, four key blood coagulation parameters were assessed, which included activated partial thromboplastin time (aPTT), thrombin time (TT), fibrinogen (FIB), and prothrombin time (PT) (Fig. 8c-f). Among these parameters, aPTT, TT, and FIB levels remained within the normal range, as indicated by the shaded gray boxes. However, it’s worth noting that PT exceeded the standard normal range even in the control group. This is a subject of debate among scholars and clinical experts concerning the safety thresholds of PT [41]. In summary, our preliminary studies have indicated the high biocompatibility of the nanomedicine for in vivo applications. However, further investigations are needed to explore aspects such as dosage, pharmacodynamics, and toxicology to enable the clinical translation of this promising technology.

## Conclusions

In summary, our study presents TPNs/ICG-cRGD as a versatile nanomedicine designed for precise thrombosis treatment, simultaneously engaging in photothermal thrombolysis and inflammation mitigation. TPNs/ICG-cRGD were efficiently synthesized and meticulously characterized, showcasing remarkable ICG loading, heightened photothermal performance and stability, robust RONS scavenging capabilities, and specific targeting to activated platelets. Upon laser irradiation, TPNs/ICG-cRGD demonstrated substantial thrombolysis potential against a variety of thrombus types, with exceptional efficacy demonstrated in an FeCl_3_-induced rat carotid artery thrombosis model. Additionally, their potent RONS-scavenging capacity exerted a pronounced anti-inflammatory effect, signified by the suppression of various cytokines, including IL-6, IL-8, CRP, and TNF-α. Considering the significance of inflammatory factors in thrombotic risk, these anti-inflammatory properties hold the potential to reduce the risk of disease recurrence. Overall, this multifunctional nanomaterial holds great promise in overcoming current limitations of thrombolytic treatments, offering a comprehensive approach that combines thrombolysis and anti-inflammatory therapy for the effective management of thrombotic diseases, marking a significant advancement in the field of thrombosis treatment.

### Electronic supplementary material

Below is the link to the electronic supplementary material.

Supplementary Material 1
