# Supplementary material for: Tea polyphenol-derived nanomedicine for targeted photothermal thrombolysis and inflammation suppression
Source: J Nanobiotechnology. 2024 Apr 3;22:146. doi: 10.1186/s12951-024-02446-z (PMC10988797; doi:10.1186/s12951-024-02446-z)
Supplement: Supplementary file 1 — Supplementary Material 1 [file 12951_2024_2446_MOESM1_ESM.docx]

**Supporting Information**

**Tea Polyphenol-Derived Nanomedicine for Targeted Photothermal Thrombolysis and Inflammation Suppression**

Hui Wang^1^, Cui Tang^2^, Yuxia Xiang^1^, Chan Zou^1^, Jianming Hu^7^, Guoping Yang* ^1, 2, 5, 6^ and Wenhu Zhou* ^2, 3, 4^

^1^. Center of Clinical Pharmacology, the Third Xiangya Hospital, Central South University, Changsha, Hunan, 410013, China

^2^. Xiangya School of Pharmaceutical Sciences, Central South University, Changsha, Hunan, 410013, China

^3^. Academician Workstation, Changsha Medical University, Changsha 410219, China

^4^. NHC Key Laboratory of Prevention and Treatment of Central Asia High Incidence Diseases, Affiliated Hospital, Shihezi University, Shihezi City, Xinjiang, 832002, China,

^5^. National-Local Joint Engineering Laboratory of Drug Clinical Evaluation Technology, Changsha, Hunan, 410000, China

^6^. Hunan Engineering Research Center for Optimization of Drug Formulation and Early Clinical Evaluation, Changsha, Hunan, 410013, China

^7^. First Department of Pathology, Affiliated Hospital, Shihezi University, Shihezi City, 832002, Xinjiang Uygur Autonomous Region, China

* Corresponding authors:

[ygp9880@126.com](mailto:ygp9880@126.com) (Guoping Yang); [zhouwenhuyaoji@163.com](mailto:zhouwenhuyaoji@163.com) (Wenhu Zhou)


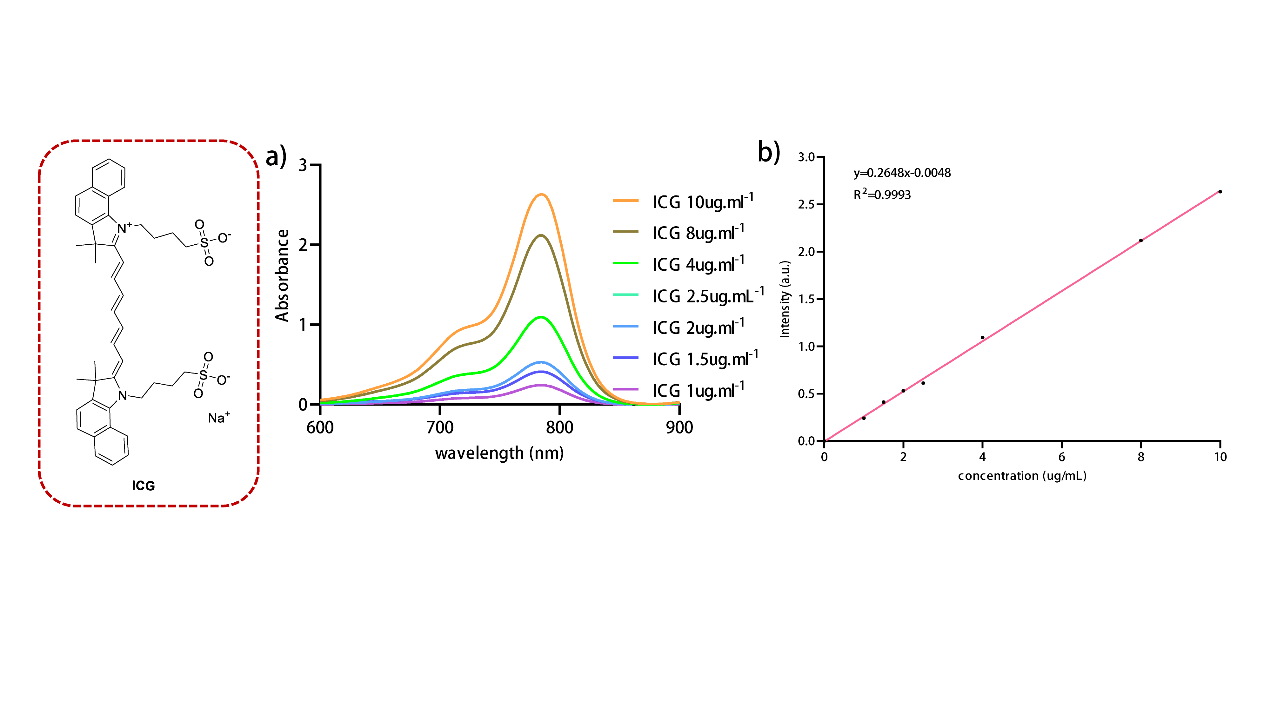


**Figure S1** a) UV-vis absorption spectra and b) standard curve of ICG.


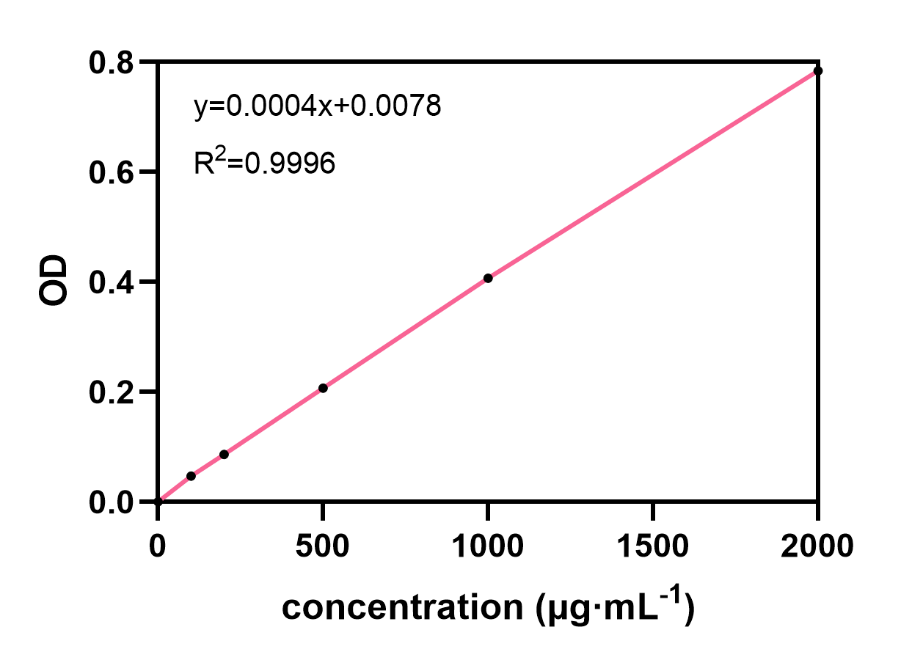


**Figure S2** The standard curve for sulfhydryl quantification.


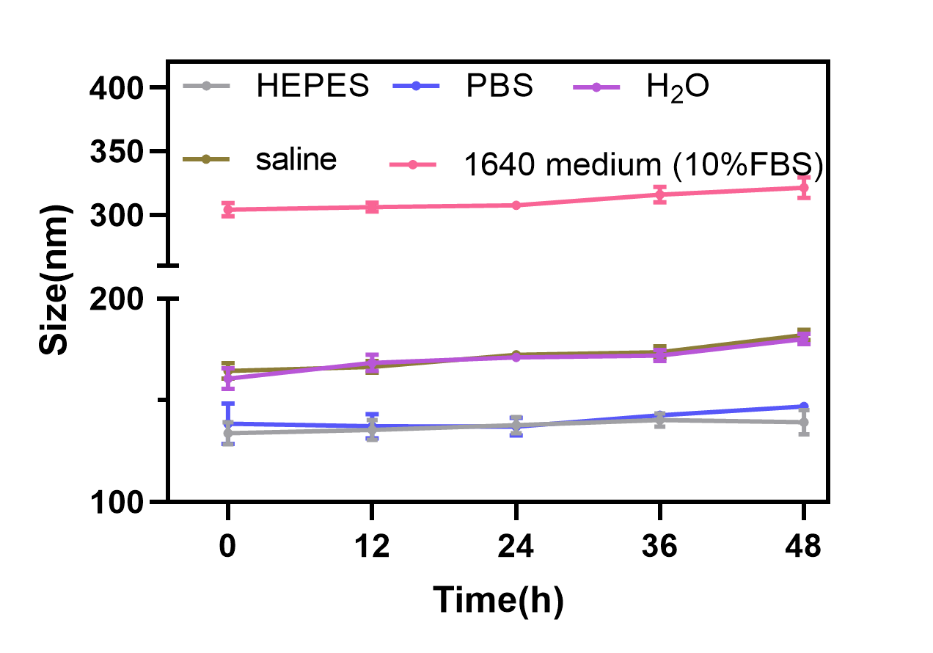


**Figure S3** The colloidal stability of TPNs/ICG-cRGD in various buffer solutions.


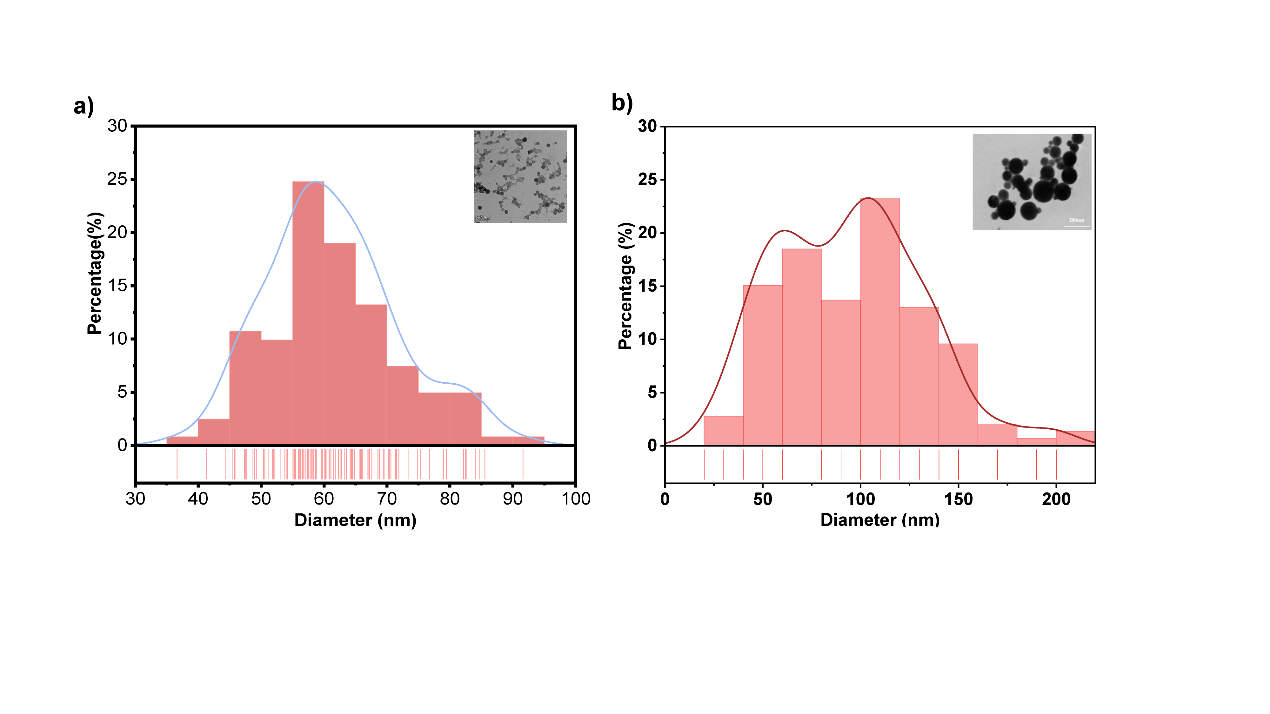


**Figure S4** The size distribution of a) TPNs, b) TPNs/ICG-cRGD obtained from TEM images. Scale bar=200 nm


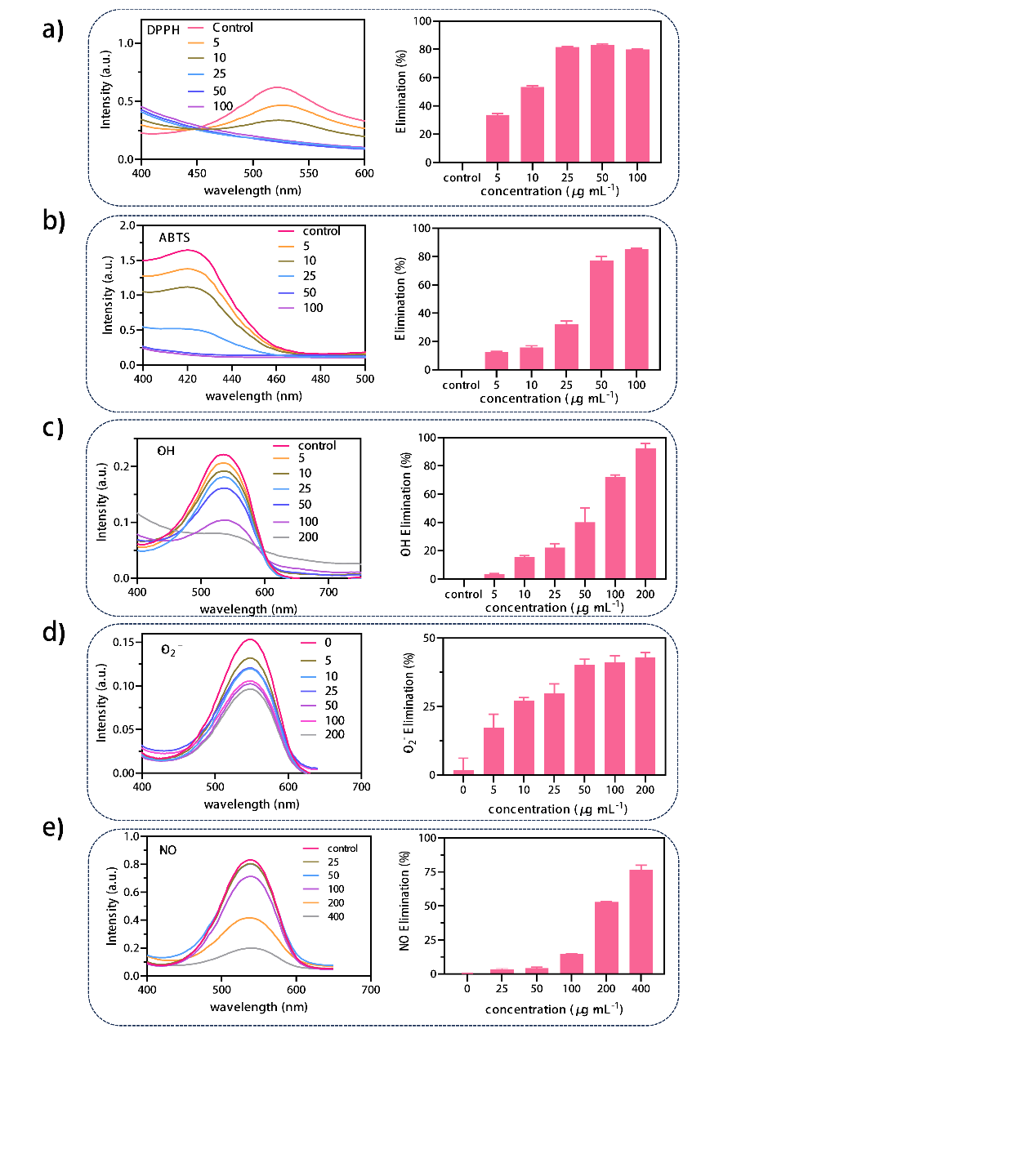


**Figure S5** UV-vis absorbance spectra of a) DPPH, b) ABTS, c) ·OH, d) ·O_2_^–^, e) ·NO, exposure to different concentrations of TPNs/ICG-cRGD, and the quantification of the RONS elimination percentage.


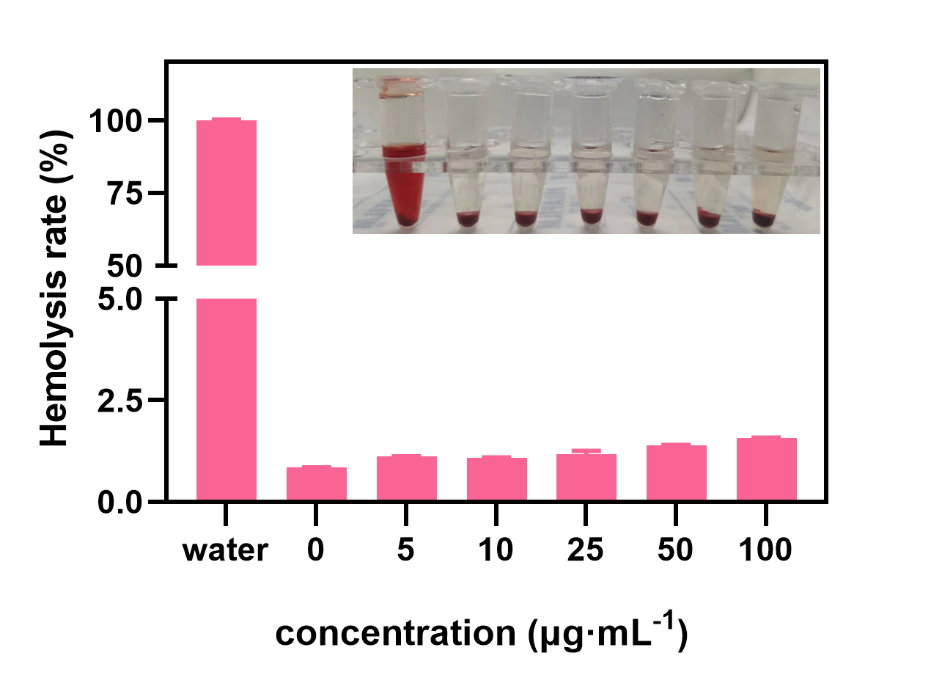


**Figure S6** Hemolysis analysis of TPNs/ICG-cRGD (n=3, mean± SD).


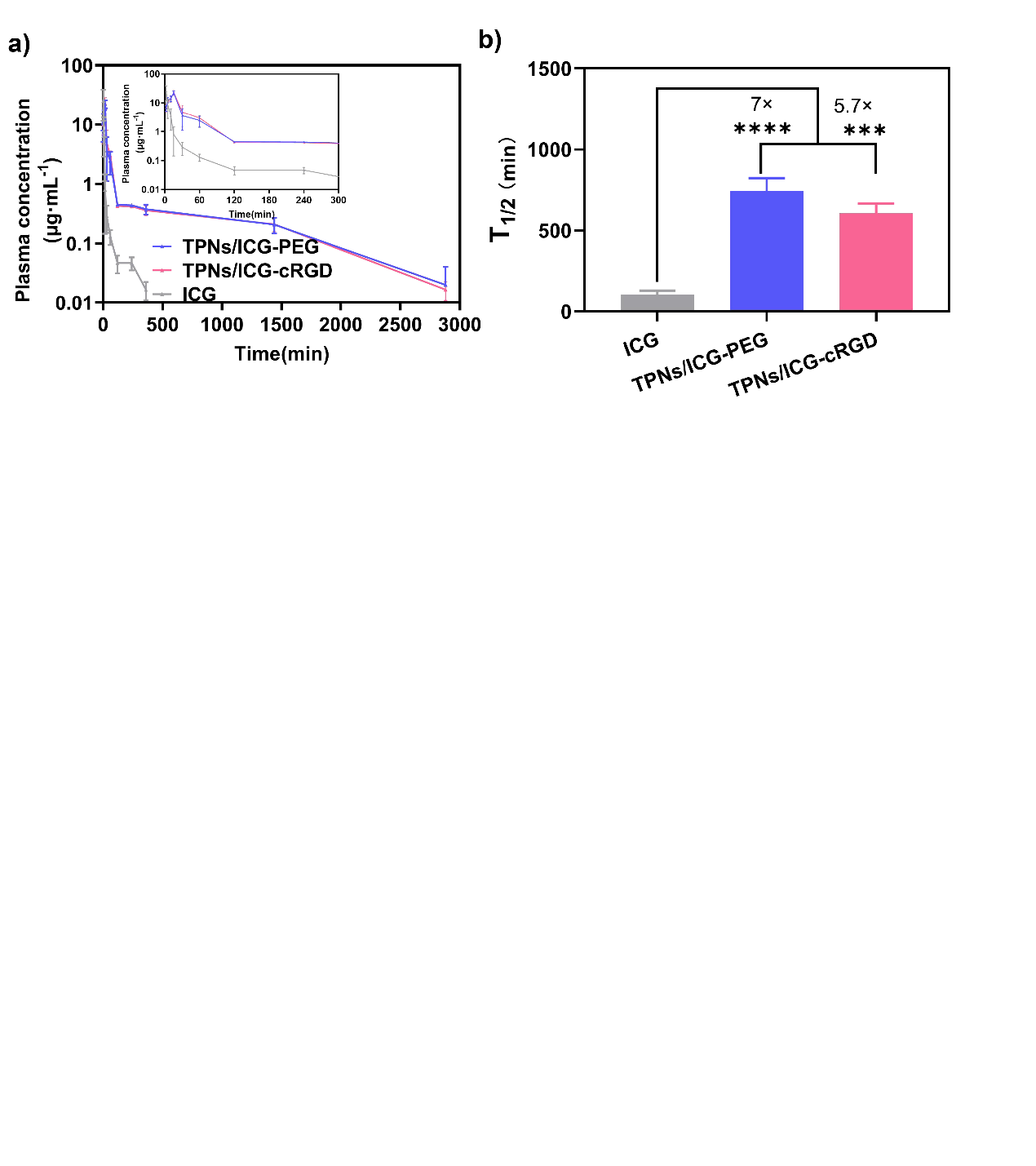


**Figure S7** a）The plasma drug concentration-time curve of different formulations (ICG, TPNs/ICG-PEG, TPNs/ICG-cRGD) after intravenous injection. b) Half-life of different formulations (ICG, TPNs/ICG-PEG, TPNs/ICG-cRGD). c) Area under the curve (AUC) of different formulations (ICG, TPNs/ICG-PEG, TPNs/ICG-cRGD). n=3, mean ± SD.


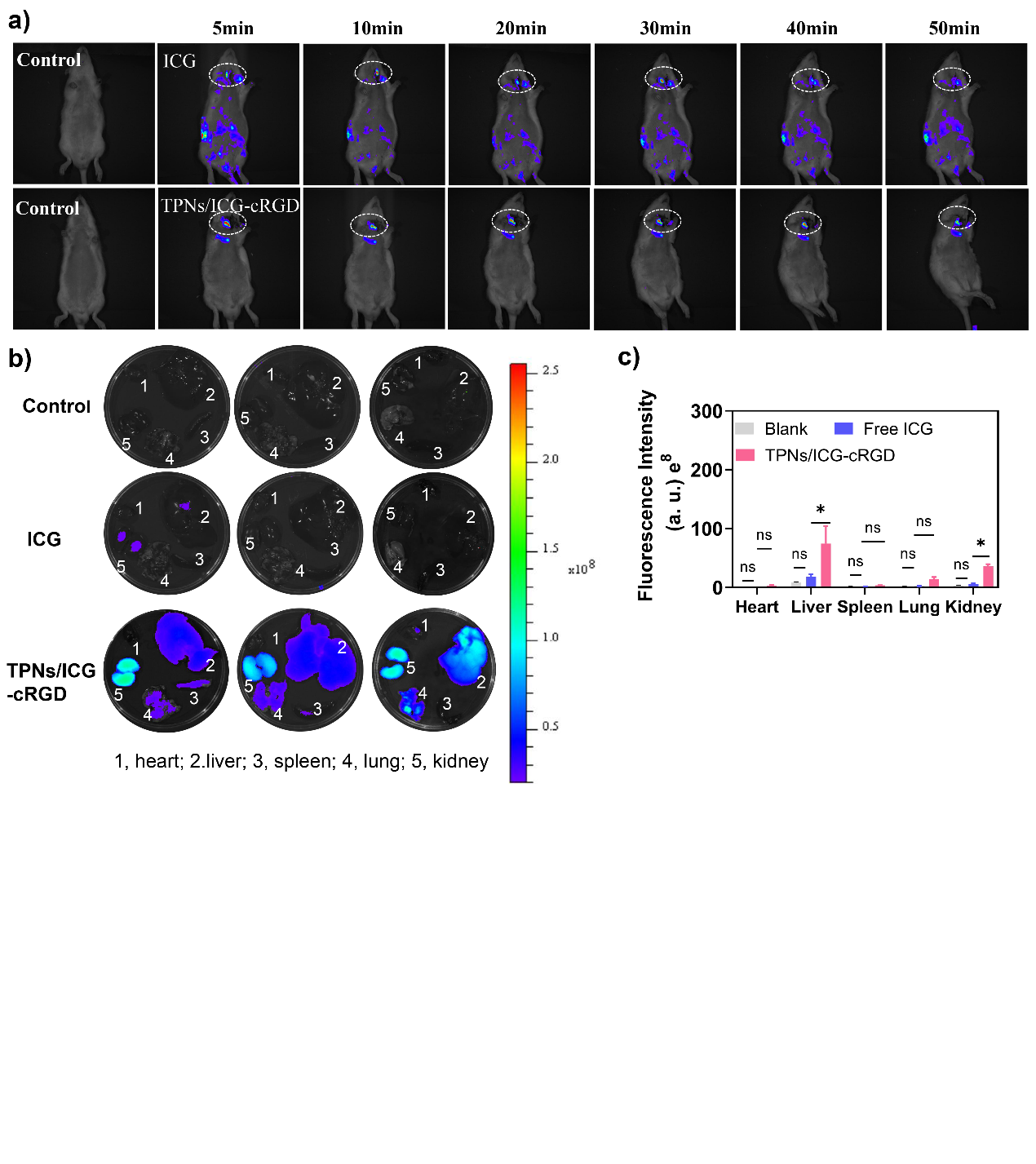


**Figure S8** a) Fluorescence images in SD rats from the ICG and TPNs/ICG-cRGD groups at different time points after injection. b) Fluorescence images of the major organs (heart, liver, spleen, lung and kidney) and c) Quantitative analysis of fluorescence intensity of organs. n=3, mean ± SD.
